# Synthesis and Antifungal Activity of Fmoc-Protected 1,2,4-Triazolyl-α-Amino Acids and Their Dipeptides Against *Aspergillus* Species

**DOI:** 10.3390/biom15010061

**Published:** 2025-01-04

**Authors:** Tatevik Sargsyan, Lala Stepanyan, Henrik Panosyan, Heghine Hakobyan, Monika Israyelyan, Avetis Tsaturyan, Nelli Hovhannisyan, Caterina Vicidomini, Anna Mkrtchyan, Ashot Saghyan, Giovanni N. Roviello

**Affiliations:** 1 Scientific and Production Center “Armbiotechnology” NAS RA, 14 Gyurjyan Str., Yerevan 0056, Armenia; 2 Institute of Pharmacy, Yerevan State University, 1 Alex Manoogian Str., Yerevan 0025, Armenia; 3 Scientific Technological Center of Organic and Pharmaceutical Chemistry, 26, Azatutian Ave., Yerevan 0014, Armenia; 4 Institute of Biostructures and Bioimaging, Italian National Council for Research (IBB-CNR), Area di Ricerca Site and Headquarters, Via Pietro Castellino 111, 80131 Naples, Italy

**Keywords:** 1,2,4-triazoles, antifungal peptides, synthesis, dipeptides, fluconazole, non-protein amino acids, *Aspergillus candidus*, *Aspergillus versicolor*, *Aspergillus flavus*, *Alternaria alternate*, * Ulocladium botrytis*, *Aureobasidium pullulans*

## Abstract

In recent years, fungal infections have emerged as a significant health concern across veterinary species, especially in livestock such as cattle, where fungal diseases can result in considerable economic losses, as well as in humans. In particular, *Aspergillus* species, notably *Aspergillus flavus* and *Aspergillus versicolor*, are opportunistic pathogens that pose a threat to both animals and humans. This study focuses on the synthesis and antifungal evaluation of novel 9-fluorenylmethoxycarbonyl (Fmoc)-protected 1,2,4-triazolyl-α-amino acids and their dipeptides, designed to combat fungal pathogens. More in detail, we evaluated their antifungal activity against various species, including *Aspergillus versicolor* (ATCC 12134) and *Aspergillus flavus* (ATCC 10567). The results indicated that dipeptide **7a** exhibited promising antifungal activity against *Aspergillus versicolor* with an IC_50_ value of 169.94 µM, demonstrating greater potency than fluconazole, a standard treatment for fungal infections, which showed an IC_50_ of 254.01 µM. Notably, dipeptide **7a** showed slightly enhanced antifungal efficacy compared to fluconazole also in *Aspergillus flavus* (IC_50_ 176.69 µM vs. 184.64 µM), suggesting that this dipeptide might be more potent even against this strain. Remarkably, **3a** and **7a** are also more potent than fluconazole against *A. candidus* 10711. On the other hand, the protected amino acid **3a** demonstrated consistent inhibition across all tested *Aspergillus* strains, but with an IC_50_ value of 267.86 µM for *Aspergillus flavus*, it was less potent than fluconazole (IC_50_ 184.64 µM), still showing some potential as a good antifungal molecule. Overall, our findings indicate that the synthesized 1,2,4-triazolyl derivatives **3a** and **7a** hold significant promise as potential antifungal agents in treating *Aspergillus*-induced diseases in cattle, as well as for broader applications in human health. Our mechanistic studies based on molecular docking revealed that compounds **3a** and **7a** bind to the same region of the sterol 14-α demethylase as fluconazole. Given the rising concerns about antifungal resistance, these amino acid derivatives, with their unique bioactive structures, could serve as a novel class of therapeutic agents. Further research into their in vivo efficacy and safety profiles is warranted to fully realize their potential as antifungal drugs in clinical and agricultural settings.

## 1. Introduction

In recent years, fungi have been recognized as integral components of commensal microbiota in various parts of the body, including the intestines, oral cavity, skin, lungs, and vagina [1,2]. They have served as a food source and played a significant role in food processing for thousands of years. Additionally, fungi may be utilized in numerous industrial processes, contributing to the production of peptides, enzymes, vitamins, organic acids, and antibiotics [3,4]. However, alongside their beneficial properties [5], fungi can act as pathogens for plants, humans, and animals. Zoonotic infections have been acknowledged for centuries and represent a significant portion of emerging and reemerging infectious diseases globally. Notably, there has been an increasing number of recalcitrant fungal diseases in animals over the last two decades, primarily stemming from opportunistic and pathogenic fungi [6,7], which can also be transmitted to humans. Opportunistic fungi have a preferred habitat independent from the living host and cause infection after accidental penetration of intact skin barriers or in the case of immunologic defects or other debilitating conditions that exist in the host [8]. In contrast, several other pathogens are characterized by their dependence on vertebrate hosts; obligate pathogens, in particular, require a host to complete their life cycle and essential functions such as nutrient acquisition, growth, habitat establishment, and reproduction [9,10].

### 1.1. Fungal Strains Affecting Animal Species

Some of the more relevant fungal strains include *Aspergillus*, *Alternaria alternata*, *Cladobotryum botrytis*, and *Aureobasidium pullulans*. Aspergillosis is an airborne fungal infection caused by molds in the genus *Aspergillus.* It primarily spreads through the inhalation of conidia, although infection can also occur via ingestion or contaminated wounds [11]. The clinical spectrum of *Aspergillus*-associated diseases in humans ranges from chronic localized aspergillomas to acute invasive aspergillosis, with severity often linked to the patient’s underlying health conditions [12,13]. In animals, the clinical manifestations of aspergillosis vary widely depending on the species and environmental factors. Here is a concise table (Table 1) summarizing how *Aspergillus* infections affect different animal species [14,15].

Fungal infections, particularly those caused by *Aspergillus* species, are a growing concern in veterinary medicine, especially in livestock such as cattle. *Aspergillus flavus* and *Aspergillus* versicolor are known opportunistic pathogens that can lead to significant health issues in animals, including mycotic abortion, respiratory infections, and gastrointestinal diseases. In cattle, *Aspergillus* infections often occur in late pregnancy, where they can cause abortion, a serious condition with major implications for both animal health and farm productivity [16]. Furthermore, these infections can lead to chronic respiratory conditions, reduced fertility, and diminished milk production, all of which contribute to considerable economic losses in the agricultural sector. In addition to direct impacts on cattle health, *Aspergillus* species are also of concern due to their ability to develop resistance to commonly used antifungal treatments. With the increasing prevalence of such infections in immunocompromised animals and the limited number of effective antifungal agents available, there is an urgent need for novel, more effective therapeutic approaches. This situation underscores the critical importance of developing targeted antifungal therapies that not only address the immediate health risks posed by *Aspergillus* species but also reduce the likelihood of resistance development, a growing challenge in both veterinary and human medicine [14,17].

*Aureobasidium pullulans* is a dematiaceous, yeast-like fungus that is ubiquitous in nature and can colonize human hair and skin. It has been implicated clinically as causing skin and soft tissue infections, meningitis, splenic abscesses, and peritonitis [18,19]. Like other saprophytic fungi, *A. pullulans* can cause disease in the setting of immunocompromised conditions [20]. *Ulocladium*, viewed as a harmless fungus, is now recognized for its potential to cause mycotoxicoses linked to contaminated crops like wheat. Its pathogenic capabilities have come to light, with reported cases of keratitis and onychomycosis in immunocompetent patients, along with skin infections in immunocompromised individuals [21,22,23]. *Alternaria species* are ubiquitous fungi known for their dark pigmentation due to melanin. They are significant plant pathogens, causing considerable economic losses in various food crops. In addition to affecting plants, Alternaria spp. can also infect animals, including warm-blooded species and humans, with increasing clinical relevance, particularly among immunocompromised patients. Their role as potent airborne allergens is noteworthy, as they contribute to allergic respiratory conditions, including severe asthma [24,25].

### 1.2. Antifungal Drugs

Four major classes of antifungal agents dominate the market: azoles, which inhibit the synthesis of ergosterol; polyenes, which interact with fungal membrane sterols physicochemically; echinocandins, which inhibit glucan synthesis; and fluorinated pyrimidines, which interfere with pyrimidine metabolism, leading to the inhibition of DNA and RNA biosynthesis [26]. 1,2,4-Triazoles are a class of heterocyclic compounds that play an important role in medicine and chemistry due to their biological and pharmacological properties; they have a wide range of biological activities such as antibacterial, antifungal, antiviral, insecticidal, and herbicidal [27,28].. This group of biologically active compounds acts by inhibiting the activity of the cytochrome P450-dependent enzyme lanosterol-14α-demethylase (CYP51), which is an important enzyme in the ergosterol biosynthesis of fungi [29]. Some azoles bind to the heme within CYP51, causing a blockade of the ergosterol biosynthesis pathway of fungi, which leads to agglomeration of 14-demethylated sterols [30]. Recently, new 1,2,4-triazole derivatives have been obtained and evaluated for fungicidal activity, and some have shown potential activity against certain fungi. In previous years, many research papers have highlighted the importance of 1,2,4-triazoles having powerful antifungal and antibacterial properties [25,31]. However, the high mortality of invasive fungal infections, the long course of treatments required, narrow spectrum activity, and cross-resistance due to similar mechanisms of action across drugs has triggered the search for safer alternatives with reduced toxicity or other enhanced features. Drug resistance development, the increasing number of immunodeficiency- and/or immunosuppression-related diseases, and limited therapeutic options available are triggering the search for novel alternatives. Recent advancements in the study of oxidative processes and xenobiotic metabolism in plants [32]., computational drug discovery methods [33], and marine-derived therapeutic innovations [34,35] highlight the growing intersection of natural substance research with modern medicine [36,37]., offering promising avenues for novel treatments. As for new antifungals, these should be less toxic for the host, with targeted or broader antimicrobial spectra (for diseases of known and unknown etiology, respectively) and modes of actions that limit the potential for the emergence of resistance among pathogenic fungi. Given these criteria, antimicrobial peptides with antifungal properties, i.e., antifungal peptides (AFPs), have emerged as powerful candidates due to their efficacy and high selectivity. These peptides have a broad spectrum of activity against bacteria, fungi, and viruses and are less likely to develop resistance compared to conventional antibiotics [38,39,40,41]. Antifungal peptides (AFPs) are typically classified based on origin: natural, semisynthetic, or synthetic [42,43]. Semisynthetic and synthetic amino acids [44] and peptides are designed to improve pharmacological properties, reduce side effects, and lower immunogenicity compared to natural peptides and proteins [45,46]. These modifications also enhance stability and bioavailability for clinical use. For example, replacing the linoleoyl side chain of echinocandin B with octyloxybenzoyl (cilofungin) or pentyloxyterphenyl (anidulafungin) reduces hemolytic activity [47]. Structure–activity relationships (SAR) help guide the design of these peptides, with key factors such as net charge, hydrophobicity, amphipathicity, and peptide length influencing antifungal activity. Hydrophobicity and amphipathicity are crucial for membrane disruption, though higher levels may increase toxicity. Short antimicrobial peptides (SAMPs) (2–10 amino acids) are gaining attention due to their lower toxicity, greater stability, and simpler synthesis [48]. Combinatorial libraries and de novo design strategies, along with targeting fungal virulence traits and multiligand molecules like dendrimers, are employed to develop more effective peptides [49].

### 1.3. Aim of This Work

In summary, synthetic and semisynthetic peptides offer significant potential for improving antifungal therapies, with better targeting, stability, and reduced toxicity compared to natural peptides [50]. When designing and synthesizing highly bioactive compounds, it is possible to synthesize hybrid molecules containing two active fragments: triazoles and peptide moieties—a new and useful strategy [51]. The combination of these two groups yields new peptides that may have the following properties:

(i) Membrane disruption: the triazole ring can insert into fungal membranes, disrupting their integrity, causing leakage of cellular contents, and leading to cell death;
(ii) Inhibition of ergosterol biosynthesis: similar to other triazole drugs, these peptides may inhibit ergosterol synthesis, a key component of fungal cell membranes, impairing membrane function;
(iii) Cell wall disruption: triazole peptides may interfere with the synthesis of fungal cell wall components (e.g., chitin, β-glucan), weakening the cell wall and causing osmotic lysis;
(iv) DNA and protein synthesis inhibition: the triazole ring can bind to metal ions involved in DNA replication and interfere with enzymes crucial for protein synthesis;
(v) Increased stability and bioavailability: the incorporation of triazole increases the peptide’s stability, bioavailability, and resistance to enzymatic degradation;
(vi) Immune modulation: some peptides may enhance immune recognition and responses, aiding in fungal clearance.

**Scheme 1:**
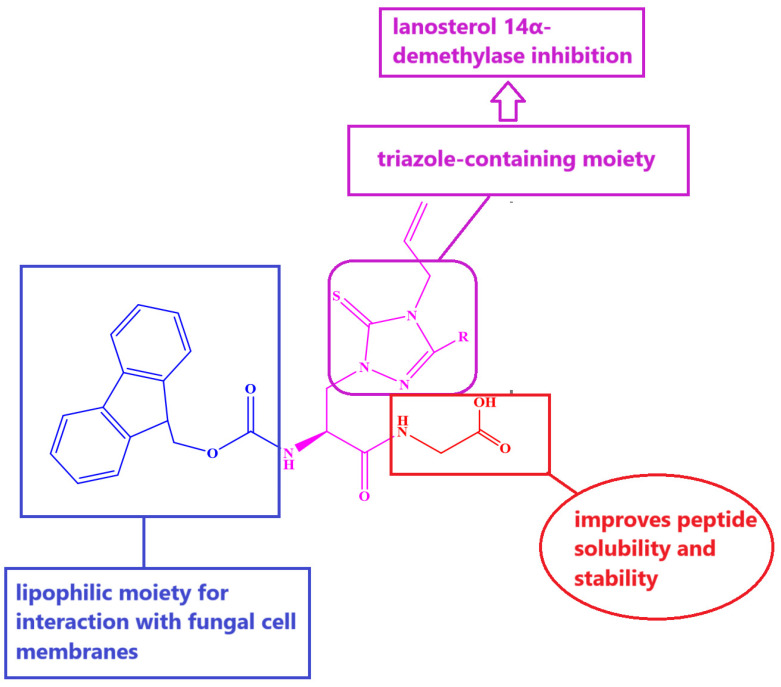
Schematic representation of the rationale for the structural moieties in our Fmoc dipeptide for antifungal properties.

Overall, triazole-containing peptides work by disrupting fungal cell structures and interfering with essential biosynthetic pathways, making them effective in combating fungal infections [52,53].. Thus, to develop new and effective fungicidal agents based on 1,2,4-triazole derivatives linked to amino acid residues, we synthesized novel dipeptides incorporating 1,2,4-triazole along with alanine and glycine. Additionally, we employed the protective group 9-fluorenylmethoxycarbonyl (Fmoc), which also possesses inherent antimicrobial activity due to its lipophilic nature [54]. In our design, the Fmoc group provides lipophilicity, enhancing interaction with the fungal membrane. The triazole moiety inhibits the fungal enzyme lanosterol 14α-demethylase, disrupting ergosterol synthesis and compromising membrane integrity. The glycine at the C-terminus imparts flexibility, minimizes steric hindrance, and potentially improves solubility and stability, supporting the compound’s overall antifungal activity (Scheme 1).

This dual functionality of Fmoc—acting both as a protective group and as an active antimicrobial component—provides a valuable strategy for developing more effective drugs [55,56], which we aimed to investigate in the context of antifungal applications, as described in the sections below.

## 2. Materials and Methods

### 2.1. Materials

The materials used in this study included dimethylsulfoxide (DMSO), DCC, N-hydroxysuccinimide, Fmoc-OSu, Gly, C_6_H_14_, C_4_H_8_O_2_, CH_3_COOC_2_H_5_, CH_2_Cl_2_, NaHCO_3_, Na_2_CO_3_, and NaOH, which were purchased from Sigma Aldrich (St. Louis, MA, USA). The non-proteinogenic amino acids (S)-β-4-allyl-3-(2-methoxyphenyl)-5-thioxo-1,2,4-triazol-1-yl]alanine and (S)-β-[4-allyl-3-(furan-2-yl)-5-thioxo-1,2,4-triazol-1-yl]alanine were synthesized at the “Armbiotechnology” Scientific and Production Center (SPC) of National Academy of Sciences of the Republic of Armenia (NAS RA). All physicochemical properties of the non-proteinogenic amino acids are described in the catalog of the “Armbiotechnology” SPC of NAS RA, available at (http://www.armbiotech.am/, accessed on 13 December 2024). All chemicals were obtained from commercial suppliers and used without further purification. All solvents were freshly distilled before use. Thin-layer chromatography (TLC) was performed on Merck aluminum foil-backed sheets precoated with 0.2 mm Kieselgel 60 F254 (Darmstadt, Germany). The proton (^1^H) and carbon-13 (^13^C) nuclear magnetic resonance (NMR) spectra were recorded on a Varian Mercury 300 MHz spectrometer, using tetramethylsilane (TMS) as the internal standard (Palo Alto, CA, USA). Melting points were determined using an electrothermal apparatus (Bibby Scientific, Stone, UK). Mass Spectrometry Analysis. Sample preparation: 0.1 mg of test substance was dissolved in 1 mL of methanol. The sample solution was filtered through a 0.45 µm syringe filter PTFE 100. Sample analysis utilized a Prominence I LC-2030C 3D Plus instrument from Shimadzu, Kyoto, Japan. The mobile phase comprised a mixture of 0.1% formic acid aqueous solution (20%) and methanol (80%). A flow rate of 0.2 mL/min was maintained, with the column temperature set at 30 °C. The injection volume stood at 0.1 µL. Detection of the sample employed a basic quadrupole MS system (LC-MS-2020, Shimadzu, Kyoto, Japan) operating in positive ionization mode via electrospray ionization (ESI). Nitrogen gas served as both the nebulizing and drying agent, with the interface temperature, heat block, and DL temperature set at 350 °C, 200 °C, and 250 °C, respectively. Data acquisition and processing were performed using Shimadzu’s LabSolutions software (version 5.99 SP2, Shimadzu, Kyoto, Japan). The *m*/*z* values of the target ions were monitored under the specified experimental conditions. IR characterization: the infrared analysis made use of an ATR (Attenuated Total Reflection) accessory, which allowed us to conduct a direct examination of the powder samples using a IRTracer-100 instrument (Shimadzu, Kyoto, Japan) using a KBr prism (4000–350 cm^−1^) with single reflection, at resolution 4 cm^−1^.

### 2.2. Synthesis of Derivatives of 9-Fluorenylmethoxycarbonyl Protected α-Amino Acids

Briefly, 0.0043 mol of the α-amino acid was dissolved in 0.0043 mol of 15% Na_2_CO_3_ and stirred at room temperature until a transparent solution formed, after which 0.0058 mol of 9-fluorenyl-methoxycarbonyl-N-oxysuccinimide ester, dissolved in 1 mL of 1,4-dioxane and 1 mL of acetone, was added to the reaction mixture. After the completion of the reaction, the reaction mixture was diluted twice with distilled water and the pH was adjusted to 6.5 with hydrochloric acid, during which the crystals of the protected amino acid precipitated at the bottom of the flask. The reaction was monitored by TLC.

As a result of the synthesis of 9-fluorenylmethoxycarbonyl-(*S*)-β-4-allyl-3-(2-methoxyphenyl)-5-thioxo-1,2,4-triazol-1-yl]-alanine, a white crystalline solid was obtained (75% yield) and exhibited a melting point of 104–106 °C. Found (%) C, 64.34; H, 4.67; N, 9.87; Calc for C_30_H_28_N_4_O_5_S (%) C, 64.73; H, 5.07; N, 10.07. ESI MS (*m*/*z*): 556.64 (found) 557.10 (expected for [C_30_H_28_N_4_O_5_S]+H^+^). ATR-IR, ν, cm^−1^: ATR-IR, ν, cm^−1^: 3869.20 (N-H); 3587.60 (O-H); 2924.09 (C–H allyl); 1693.50 (C=O, ester), 1681.93 (C=O acid); 1608.63 (C=O carbamate); 1519.91 (C=N carbamate); 1485.19 (C=C aromtic); 1442.75 (C=S), 1330.88 (-OCH_3_); 1284.59 (C-N); 1219.01 (C-N); 1037.70 (C–N heterocyclic).

^1^H NMR spectra (DMSO/CCl_4_ 1/3, δ, p.p.m, Hz): 10.9 (1H, COOH); 7.75–7.71 m (2H, Ar); 7.68–7.61 m (2H, Ar); 7.50–7.43 m (1H, Ar); 7.41 b.d (1H, J = 8,2, NH); 7.37–7.19 m (5H, Ar); 7.03–6.99 m (1H, Ar); 6.96–6.90 m (1H, Ar); 5.64 ddt (1H, J = 17.1, 10.5, 5.5, =CH); 4.94 br.d (1H, J = 10.5, CH_2_); 4,80 br.d (1H, J = 17.1, =CH_2_); 4.75–4.68 m (2H); 4.55–4.46 m (3H); 4.29–4.11 m (3H, OCH_2_CH); 3.75 s (3H, CH_3_): ^13^C: 170.5; 166.9; 156.8; 155.2; 147.8; 143.6; 143.4; 140.5; 140.4; 132.1; 131.4; 130.7; 127.0; 126.9; 126.5; 125.1; 125.0; 120.1; 119.3; 119.2; 117.0; 114.4; 110.9; 65.9 (OCH_2_); 55.1 (OCH_3_); 52.1 (NCH); 48.9 (CH_2_); 46.7 (CH_2_); 46.5 (CH).

As a result of the synthesis of 9-fluorenylmethoxycarbonyl-(*S*)-β-[4-allyl-3-(furan-2-yl)- 5-thioxo-1,2,4-triazol-1-yl]-alanine, a white crystalline solid was obtained (73% yield) and exhibited a melting point of 140–142 °C. Found (%) C, 62.81; H, 4.82; N, 10.38; Calc for C_27_H_24_N_4_O_5_S (%) C, 62.78; H, 4.68; N, 10.85. ESI MS (*m*/*z*): 516.57 (found) 517.00 (expected for [C_27_H_24_N_4_O_5_S]+H+). ATR-IR, ν, cm^−1^: 3734.19 (NH); 3491.16 (OH); 3066.82 (C-H aromatic); 2924.09 (C–H allyl); 1712.79 (C=O, acid), 1624.06 (C=O carbamate); 1523.76 (C=N carbamate); 1354.03 (C=S), 1334.74 (C–H); 1172.72 (C–O ether), 1103.28 (C–N); 1076.28 (C–N heterocyclic); 1049.28 (C–N).

^1^H NMR spectra (DMSO/CCl_4_ 1/3; δ, p.p.m, Hz): 7.44 br.d (J = 7.9, NH); 6.92 dd (J = 3.5, 0.8); 6.51 dd (J = 3.5, 1.8); 5.89 ddt (J = 17.1, 10.6, 5.1); 5.14 br.t (J = 10.6); 5.11 br.d (J = 17.1); 4.97–4.83 m; 4.78–4.55 m (2H); 4.46 m (3H); 4.21–4.10 m (3H, OCH_2_CH): ^13^C: 170.4; 167.4; 155.3; 144.3; 143.6; 143.4; 141.4; 140.5; 140.4; 139.7; 130.6; 126.9; 126.5; 125.1; 125.0; 119.2; 117.2 (=CH_2_); 112.4 (C-4 fur); 111.4 (C-4 fur); 65.9 (OCH_2_); 51.8 (NCH); 49.3 (NCH_2_); 47.0 (NCH_2_); 46.5 (CH); 40.3 (NCH_2_).

### 2.3. Synthesis of N-Oxysuccinimide Esters

Briefly, 1.75 g (8.5 mmol) of DCC previously dissolved in 3 mL of dioxane was added to a solution of (7.9 mmol) of derivatives of 9-fluorenylmethoxycarbonyl protected α-amino acid and 0.95 g (8.2 mmol) of N-hydroxysuccinimide in a mixture of 1.0 mL of dioxane and 2.7 mL of methylene chloride at 0 °C. The reaction mixture was stirred for 2 h at 0 °C and left overnight in the refrigerator. The reaction progress was monitored by TLC (chloroform/ethyl acetate/methanol, 2:4:1). The resulting precipitate of dicyclohexylurea (DCU) was filtered, and the filtrate containing the intermediate product succinimide ester of the amino acid was concentrated under vacuum to form an oily mass. As a result, stable intermediate compound **3a** was synthesized in 70% yield and **3b** in 75% yield, which were immediately used in the synthesis of peptides.

### 2.4. Synthesis of Dipeptides

Briefly, 0.085 g (1 mmol) of NaHCO_3_ was introduced into a solution of 0.08 g (1.5 mmol) of glycine in 2 mL of 0.5 M NaOH, and then 1.6 mmol of N-hydroxysuccinimide ester of 9-fluorenylmethoxycarbonyl protected α-amino acids in 4 mL of dioxane was added. The reaction mixture was stirred for 3 h at room temperature, then transferred to a separatory funnel and 6 mL of ethyl acetate, 3 mL of 10% citric acid solution, and 0.2 g of NaCl were added. After intense stirring, the organic layer was separated and dried with magnesium sulfate and the solvent was distilled off under vacuum at 50 °C. The residual matter was crystallized from a mixture of ethyl acetate and petroleum ether in a ratio of 1/3. As a result, **4a** (yield 65%) and **4b** (yield 60%) dipeptides were synthesized. The dipeptide 9-fluorenylmethoxycarbonyl-(S)-β-[4-allyl-3-(2-methoxyphenyl)-5-thioxo-1,2,4–triazol-1-yl]-α-alanylglycine is a white crystalline substance with a melting point of 182–183 °C. Found (%) C, 62.29; H, 5.39; N, 11.61; Calc for C_32_H_31_N_5_O_6_S (%) C, 62.63; H, 5.09; N, 11.41. ESI MS (*m*/*z*): 613.69 (found) 614.01 (expected for [C_32_H_31_N_5_O_6_S]+H^+^). ATR-IR, ν, cm^−1^: 3869.20 (NH); 3649.32 (NH); 3587.6 (OH); 3066.82 (C-H arom); 1716.65 (C=O, acid), 1693.50 (C=O eter); 1616.35 (C=O carbamate); 1581.63 (C=N carbamate); 1535.34 (C=C, aromatic); 1431.18 (C=S), 1365.6 (-OCH_3_); 1334.74 (C–O); 1296.16 (C–N); 1234.44 (C–N); 1118.71 (C–N heterocyclic); 1083.9 (C–N heterocyclic).

^1^H NMR spectra (DMSO/CCl_4_ 1/3, δ, p.p.m, Hz): 12.0 v.b. (1H, COOH); 8.16 br.t (1H, J = 5.4, NHCH_2_); 7.75–7.70 m (2H, Ar-H and NH); 7.68–7.61 m (2H); 7.48–7.19 m (7H); 7.50–7.40 m (2H); 7.01–6.88 m (2H); 5.69–5.54 m (1H, =CH Ally); 4.93–4.73 m (3H); 4.65–4.45 m (4H); 4.28–4.06 m (3H, OCH_2_CH); 3.82 b.d. (2H, J = 5.4, CH_2_NH); 3.71 s (3H, CH_3_): 170.3; 168.5; 166.9; 156.8; 155.2; 147.9; 143.6; 143.4; 140.5; 140.4; 132.1; 131.4; 130.7; 127.0; 126.9; 126.5; 125.2; 125.0; 120.1; 119.23; 119.19; 116.9; 114.4; 110.9; 66.1 (OCH_2_); 55.0; 53.4 (OCH_3_); 49.6; 46.7 (NCH_2_); 46.5 (CH); 40.7 (NCH_2_).

The dipeptide 9-fluorenylmethoxycarbonyl-(*S*)-β-[4-allyl-3-(furan-2-yl)-5-thioxo-1,2,4-triazol-1-yl]-α–alanylglycine is a white crystalline substance with a melting point of 169–170 °C. Found (%) C, 60.85; H, 5.1; N, 12.36; Calc for C_29_H_27_N_5_O_6_S (%) C, 60.72; H, 4.74; N, 12.21. ESI MS (*m*/*z*): 573.62 (found) 574.05 (expected for [C_29_H_27_N_5_O_6_S]+H^+^). ATR-IR, ν, cm^−1^: 3649.32 (NH); 3630.03 (NH); 3471.87 (OH); 2927.94 (C–H allyl); 1701.22 (C=O, acid), 1581.63 (C=O carbamate); 1519.91 (C=N carbamate); 1446.61 (C=C, aromatic); 1354.03 (C=S), 1172.72 (C–O, ether), 1122.57 (C–O, acid); 1103.28 (C–N); 1045.42 (C–N).

^1^H NMR spectra (DMSO/CCl_4_ 1/3, δ, p.p.m, Hz): 8.17 br.t (1H, J = 5.6, CH_2_NH); 7.74–7.70 m (2H, Ar.); 7.68–7.58 m (3H, Ar + H-5 fur.); 7.74–7.70 m (2H, Ar.); 7.39 br.d. (1H, J = 9.1, NHCH); 7.36–7.30 m (2H, Ar.); 7.28–7.21 m (2H, Ar.); 6.90 br.d. (1H, J = 3.3, H-3 fur.); 6.48 dd (1H, J = 3.3, 1.8, H-4 fur.); 5.87 ddt (1H, J = 16.6, 11.1, 5.0, =CH); 5.15–5.05 m (2H, =CH_2_); 4.95–4.81 m (2H, CH_2_, All); 4.77 td (1H, J = 9.1, 4.1); 4.63 dd (1H, J = 13.7, 4.1); 4.47 dd (1H, J = 13.7, 9.4); 4.29–4.03 m (3H); 3.83 dd (1H, J = 18.0, 5.6, CH_2_NH); 3.81 dd (1H, J = 18.0, 5.6, CH_2_NH).

^13^C: 170.4; 168.5; 167.5; 155.3; 144.3; 143.6; 143.4; 141.5; 140.5; 140.4; 139.7; 130.6; 127.0; 126.5; 125.2; 125.0; 119.3; 117.1 (=CH_2_); 112.5 (C-3 fur.); 111.4 (C-4 fur.); 66.1 (OCH_2_); 53.2 (NCH); 50.0 (NCH_2_); 47.0 (NCH_2_); 46.5 (CH); 40.7 (NCH_2_).

### 2.5. Antifungal Activity Assessment

In our work, the antifungal activity of the synthesized compounds was evaluated against various fungal strains: *Aspergillus versicolor* 12134, *Aspergillus flavus* 10567, *Aspergillus candidus* 10711, *Alternaria alternata* 8126, *Ulocladium botrytis* 12027, and *Aureobasidium pullulans* 8269. The compounds were dissolved in DMSO to prepare 0.05 M solutions and tested at three different concentrations (89 μM, 183 μM, 278 μM) in 90 mL Czapek medium. Each sample was tested in triplicate across Petri dishes, with three strains per dish. The Petri dishes were incubated at 28 °C for 5–7 days. Fungal growth was assessed visually and with the aid of a magnifying glass. The intensity of fungal growth was compared to control plates to evaluate the antifungal effect of the compounds. To obtain comparative data during the research, the obtained data were compared with those obtained using a fluconazole solution of the same molarity. The data are presented in the Supporting Information and Results and Discussion sections. The histograms related to fungal growth inhibition were obtained by analyzing colony intensities using ImageJ (Rasband, W.S., U.S. National Institutes of Health, Bethesda, MD, USA, https://imagej.nih.gov/ij/, accessed on 13 November 2024). ImageJ is an open-source image analysis software widely used in scientific research for digital image processing and intensity quantification. For data collection, the plates with fungal colonies were compared before and after treatment with the compounds isolated and described in our manuscript. The procedure was performed three times in different areas of the analyzed images to ensure adequate representation of the results. The intensity values were provided as the mean ± standard deviation (SD = ±2–10%). This methodology was chosen as a modification of a standard method (CLSI M38-A2, reference protocol for antifungal testing).

### 2.6. Molecular Docking

The fungal sterol 14-α demethylase model used in our simulations corresponds to the structure with PDB ID: 5F3B, which was visualized using Discovery Studio (DS) 2021 software (Accelrys, San Diego, CA, USA) [57], with the ligand removed manually. The complex predictions were obtained by docking the ligands fluconazole, **3a**, and **7a** with the target protein using the HDOCK software (http://hdock.phys.hust.edu.cn/, accessed on 1 November 2024), [58], with default parameters. The ligand structures were generated using the MolView program (https://molview.org/, accessed on 1 November 2024, The Netherlands, v2.4), which, after energy minimization, allowed us to create three-dimensional models saved as pdb files and visualized in DS. HDOCK, which is suitable for both macromolecule–macromolecule and small molecule–macromolecule docking [54,55,56,57,58,59], was used for the blind docking described in this study. The software employs the iterative knowledge-based scoring function ITScore-PP to rank the top 10 poses obtained after the docking simulations. The HDOCK score, which is an energy score represented as a dimensionless value, indicates the binding strength between the molecules, with larger negative values reflecting stronger interactions. This scoring method has been shown to correlate well with experimental binding affinities [60]. The top-ranked pose (top 1) and the top 1 to top 3 poses for the complexes predicted by HDOCK are considered in our analysis.

## 3. Results

### 3.1. Synthesis of Dipeptides

**Scheme 2:**
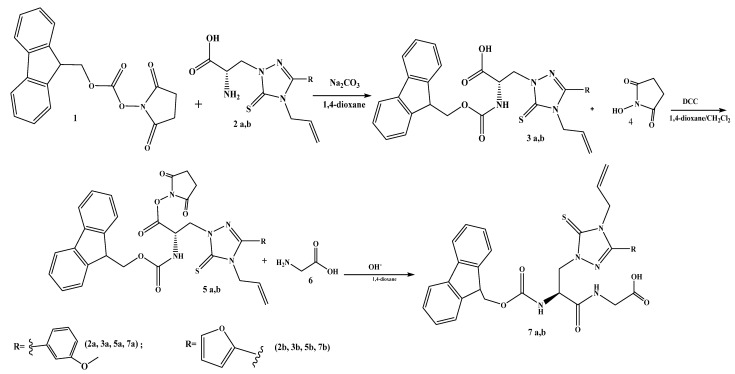
Schematic representation of the synthesis pathway for the dipeptides **7a** and **7b**.

The synthesis of dipeptides **7a** and **7b** containing non-protein amino acids **2a** and **2b** was carried out according to Scheme 2. In the first stage, the protected amino acids 9-fluorenylmethoxycarbonyl (Fmoc)-protected alanine (**3a**, **3b**) were synthesized. The reaction was carried out in a slightly alkaline medium at room temperature (Scheme 2) according to a previously published procedure [61], the experimental conditions of which were optimized by us in the present work. After testing the solvents acetone, isopropanol, and dioxane, we came to the conclusion that the best option for protecting the triazole-containing amino acids was a solvent mixture in an equal ratio of 1,4-dioxane/acetone.

When working up the reaction mixture, it was not possible to get rid of the excess Fmoc-N-hydroxysuccinimide ester and the resulting N-hydroxysuccinimide using the standard method, since they are almost equally soluble in both diethyl ether and ethyl acetate. An accessible and effective method was developed for processing the reaction mixture. By adding water and adjusting the pH to 5.5 with 0.1 N HCl, excess Fmoc-N-hydroxysuccinimide ester was removed, causing the protected amino acid to precipitate immediately at the first stage. This is explained by the fact that the solubility of 9-fluorenylmethoxycarbonyl-N-hydroxysuccinimide ester in water is higher than the solubility of the protected amino acids. Subsequently, a small amount of residual **1** was removed using diethyl ether, since this ester was more soluble in diethyl ether than the protected amino acids. During the neutralization of the reaction mixture, the molarity of hydrochloric acid was adjusted, since concentrated hydrochloric acid could cause unwanted transformations or reactions of the synthesized compounds. The optimal concentration was 0.1 N, at which the protected amino acids remained stable. The subsequent step was the transformation of the Fmoc-amino acids into succinimide esters. This was achieved by activating the carboxyl groups of the Fmoc-amino acids with N-hydroxysuccinimide (HOSu) (**4**) in the presence of dicyclohexylcarbodiimide (DCC) as a coupling reagent. The reaction was carried out in a solvent mixture of dioxane and methylene chloride, resulting in N-hydroxysuccinimide esters **5a** and **5b** [62]. In the next step, glycine (6) was condensed with N-hydroxysuccinimide esters of the Fmoc-amino acids **5a** and **5b**. The condensation reactions were carried out in different media using different molar ratios of sodium hydroxide and sodium carbonate. Optimal results were achieved with a NaOH/Na_2_CO_3_ molar ratio of 2:1. Reactions carried out with NaOH alone, in the absence of Na_2_CO_3_, resulted in increased formation of by-products. Thus, the dipeptides **7a** and **7b** were successfully synthesized, as shown in Scheme 2.

### 3.2. Antifungal Activity

An in vitro study was conducted to evaluate the antifungal effects of fluconazole, the synthesized amino acids, and their corresponding dipeptide derivatives, with the aim of obtaining comparative data. The following fungal strains were selected as study subjects: *Aspergillus versicolor* 12134, *Aspergillus flavus* 10567, *Aspergillus candidus* 10711, *Alternaria altenata* 8126, *Ulocladium botrytis* 12027, *Aureobasidium pullulans* 8269. The influence of different concentrations of all samples (89 μM, 183 μM, and 278 μM) on the activity of the aforementioned fungi was studied.

**Figure 1:**
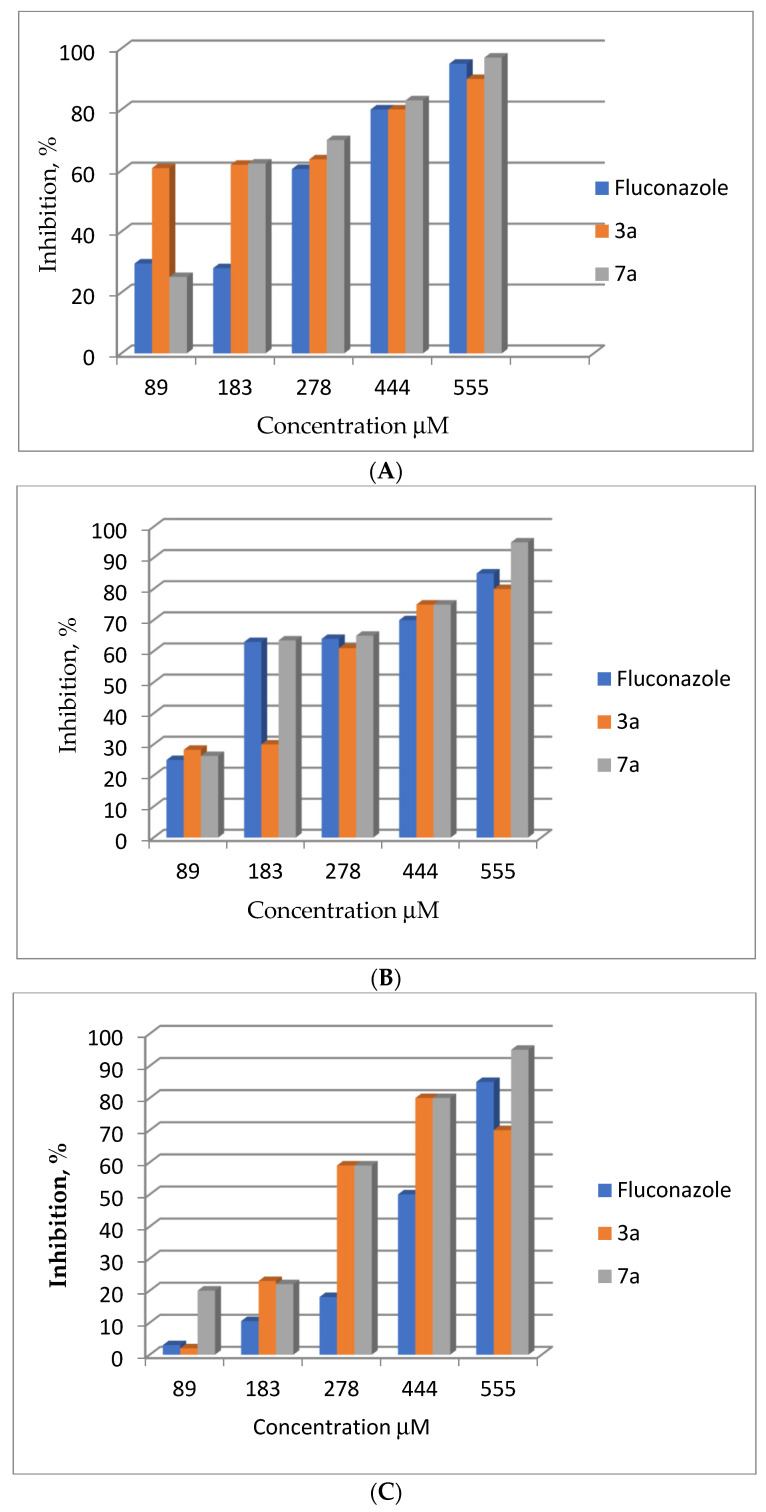
Histograms showing the antifungal effects of compounds **3a**, **7a**, and fluconazole on *A. versicolor* 12134 (**A**), *A. flavus* 10567 (**B**), and *A. candidus* 10711 (**C**). For photographs of the fungal plates treated with the different compounds, please refer to Figure S1 in the Supporting Information. The X-axis indicates the concentrations of fluconazole and compounds **3a** and **7a** in the cultures.

The results are depicted in Figure S1, which illustrates the observed antifungal activities based on visual appearance, and in Table 2, which lists the antifungal activities of the three different concentrations of the compounds and the reference fluconazole against the selected fungal strains. Taking into account the above data, the percentage of inhibition of strains *Aspergillus versicolor* 12134, *A. flavus* 10567, and *A. candidus* 10711 by the two most active compounds (**3a** and **7a**) was calculated and compared to fluconazole (Figure 1A–C). Table 2 presents the antifungal activity of fluconazole and the test compounds (**2a**, **2b**, **3a**, **3b**, **7a**, **7b**) at varying concentrations against the six fungal strains. These strains are identified by their corresponding strain numbers, as registered in the Microbial Depository Center and include *Aspergillus versicolor* 12134, *A. flavus* 10567, *A. candidus* 10711, *Alternaria alternata* 8126, *Ulocladium botrytis* 12027, and *Aureobasidium pullulans* 8269. The data are shown for the above-mentioned three different concentrations (89 μM, 183 μM, and 278 μM), with the antifungal response indicated as follows.

For each concentration, the presence or absence of antifungal activity was recorded for each strain, with higher concentrations generally leading to more pronounced antifungal effects. The experiments revealed that several samples had an effect on the selected strains. When comparing the numerical data to fluconazole, dipeptide **7a** at a dose of 89 μM was found to suppress the growth of *Aspergillus versicolor* 12134 and *A. flavus* 10567 strains with the same effectiveness as fluconazole (Figure 1A,B). An increase in the amount of fluconazole to 183 μM does not change the suppressive action on *Aspergillus versicolor*, while in the case of the dipeptide, the suppressive action increases significantly. In the case of 278 μM, the suppressive action of fluconazole practically coincides with the suppression of dipeptide in the case of 183 μM, and the noteworthy suppressive effects caused by the protected amino acid **3a** at concentrations of 89, 183, and 278 μM on *Aspergillus versicolor* are not significantly different from each other and are of the same order of magnitude as the suppression observed with 278 μM of fluconazole. In the case of *A. candidus* 10711, fluconazole is generally a weaker inhibitor compared to compounds **3a** and **7a** (Figure 1C).

Additionally, 278 μM of compound **3a** shows the same activity as 278 μM of compound **7a**. This suggests that compounds **3a** and **7a** exhibit similar effects at this concentration and are more effective than fluconazole *A. candidus* 10711 (Figure 1C and Figure S1). In the case of the strains different from *Aspergillus*, the effect of fluconazole is more pronounced than that of the test compounds, but a certain inhibitory effect is still present. To obtain more quantitative and comparative data, additional experiments were conducted with *Aspergillus* species using fluconazole, as well as compounds **3a** and **7a**. While initial assays were performed at 89 µM, 183 µM, and 278 µM, these were subsequently extended to higher concentrations of 444 μM and 555 μM. The inhibition percentages observed at 555 μM and the corresponding apparent IC_50_ (Figure S2) values for each substance are summarized in Table 3. Although complete (100%) inhibition was not achieved, significant partial inhibition was observed, further demonstrating the compounds’ strong antifungal activity, as shown in Figure S1. The findings revealed that dipeptide **7a** exhibited notable antifungal activity against *Aspergillus versicolor*, with an IC_50_ of 169.94 µM, indicating higher potency than fluconazole, a widely used antifungal agent, which had an IC_50_ of 254.01 µM. Interestingly, dipeptide **7a** demonstrated slightly improved antifungal effectiveness compared to fluconazole in *Aspergillus flavus* too (IC_50_ 176.69 µM vs. 184.64 µM), highlighting its potential efficacy against this strain. Moreover, the protected amino acid **3a** showed consistent inhibitory activity across all tested strains. However, with an IC_50_ of 267.86 µM for *Aspergillus flavus*, it was less potent than fluconazole (IC_50_ 184.64 µM), though it still holds promise as a viable antifungal compound. Remarkably, **3a** and **7a** are also more potent than fluconazole against *A. candidus* 10711 (Table 3).

### 3.3. In Silico Exploration of the Mechanism Behind the Activity of Compounds 3a and 7a Against Aspergillus Species: Molecular Docking of the Antifungal Molecules with Sterol 14- α Demethylase

Given the particularly interesting antifungal properties of compounds **3a** and **7a**, especially their pronounced effects on *Aspergillus* species, we sought to investigate the underlying mechanism of action responsible for this observed activity. To explore this, we selected one of the key proteins involved in fungal activity whose inhibition is linked to the action of triazole-containing antifungal drugs. Specifically, we focused on the sterol 14-α demethylase from *Aspergillus* species [63], a well-known target for azole-based antifungals like fluconazole [64].

**Figure 2:**
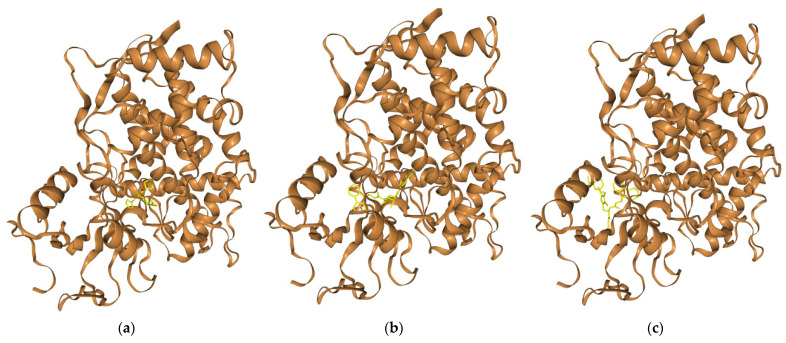
Molecular docking complex views (top 1 pose) of PDB ID 5FRB, sterol 14-α demethylase (unliganded) in complex with fluconazole (**a**), compound **3a** (**b**), and compound **7a** (**c**) visualized using the HDOCK software. The ligand is highlighted in yellow color.

To study the interaction of fluconazole and our test compounds (**3a** and **7a**) with this target protein, we performed molecular docking simulations using the HDOCK docking software [54,65]. Blind docking simulations were carried out on the protein target (PDB ID 5FRB [61]), which represents the sterol 14-alpha demethylase from an *Aspergillus* species, using fluconazole as a reference ligand, alongside the .pdb files for compounds **3a** and **7a**. As shown in Figure 2, our docking results revealed that both compound **3a** and compound **7a**, but especially compound **3a**, bind to a similar region of the protein as fluconazole. This similarity is visually confirmed by the overlap in the binding locations of the ligands, which are depicted in yellow for clarity (Figure 2). A more detailed analysis, shown in Table 4, compares the receptor interface residues common between the fluconazole–protein complex and those formed with compounds **3a** and **7a**. While the fluconazole and compound **7a** complexes share four common interface residues (TYR 122, ILE 373, LEU 503, PHE 504), compound **3a** interacts with a larger number of common residues: TYR 122, LEU 125, THR 126, TYR 136, ALA 307, GLY 308, SER 311, ILE 373, LEU 503, PHE 504, and HEM 580. The interaction diagrams for the complexes of **3a**, **7a**, and fluconazole with the protein are also shown in the Supporting Information (Figure S3). In terms of binding affinity, both compounds **3a** and **7a** demonstrate comparable, or slightly stronger, binding than fluconazole.

The HDOCK scores (both top 1 poses and the averages across poses 1–3) indicate that fluconazole binds with slightly lower affinity to the sterol 14-α demethylase than either of the test compounds, **3a** and **7a** (−193.30, 183.27 ± 11.13 vs. −207.97, 192.63 ± 11.86 and −206.62, −190.42 ± 11.51, respectively. Table 4).

This finding supports the hypothesis that compounds **3a** and **7a** may exert their antifungal effects through a mechanism similar to fluconazole, leading to similar or slightly enhanced binding to the target protein, which contributes to its antifungal activity, particularly against *Aspergillus* species as experimentally found.

## 4. Discussion

The synthesis of Fmoc-amino acids and their conversion into peptides [66,67], including dipeptides, highlights the effective use of optimized methods in peptide chemistry. The careful selection of solvent systems is essential for achieving high chemical yields during the reaction process. Selecting a solvent mixture of 1,4-dioxane and acetone in a 1:1 ratio for the protection of triazole-containing amino acids proved effective. This solvent system not only facilitated the protection of the amino acids but also contributed to the overall chemical yield.

The difficulty in separating excess 9-fluorenylmethoxycarbonyl-N-oxysuccinimide ester highlighted the common challenges encountered in solution-phase synthesis of peptides [68]. The development of a new purification strategy by adjusting the water content to exploit solubility differences was an innovative solution, emphasizing the importance of process optimization in synthetic peptide chemistry. Additionally, regulating the concentration of hydrochloric acid during neutralization was crucial. The observed degradation of synthesized compounds in the presence of concentrated acid underscores the sensitivity of these reactions to pH. Since the use of concentrated acid led to the decomposition of final products and the formation of side compounds, experimental results identified 0.1N hydrochloric acid as the optimal condition. The transition from Fmoc-amino acids to dipeptides involved activating the carboxyl groups with N-hydroxysuccinimide and dicyclohexylcarbodiimide, enabling efficient coupling with glycine. The variation in molar ratios of sodium hydroxide and sodium carbonate significantly influenced the reaction outcome. An optimal 2:1 (NaOH/Na_2_CO_3_) ratio minimized by-product formation, underscoring the importance of selecting appropriate base conditions in peptide coupling reactions. The successful synthesis of dipeptides **7a** and **7b** using this approach highlights the robustness of the employed methods and their scalability potential. The herein presented antifungal study revealed that synthesized compounds, especially dipeptides **3a** and **7a**, comparable to fluconazole, exhibited significant antifungal activity against various strains, particularly against *Aspergillus versicolor* and *A. flavus* (Table 2, Figure S1 and Figure 1). The concentration-dependent effects highlighted the importance of dosage, with dipeptide 8 showing increased efficacy at higher concentrations. Given the pronounced antifungal effects of compounds **3a** and **7a**, particularly against *Aspergillus* species, we investigated their underlying mechanism of action. We focused on the sterol 14-α demethylase [69] from *Aspergillus*, a well-known target of azole-based antifungals like fluconazole. To study the interaction of fluconazole, compound **3a**, and compound **7a** with this target, we conducted molecular docking simulations using HDOCK software, targeting the protein structure (PDB ID 5FRB) from an *Aspergillus* species. More in detail, we removed the ligand VT-1598 [70] from the structure of sterol 14 α-demethylase (CYP51B) of *Aspergillus fumigatus* [71], which was then used in the docking studies. Our docking results revealed that compounds **3a** and **7a** bind to a similar region of the sterol 14-α demethylase as fluconazole. Notably, compound **3a** displayed a broader interaction profile, engaging more receptor interface residues than fluconazole or compound **7a**. Binding affinity analysis showed that compounds **3a** and **7a** exhibited slightly stronger or comparable binding to the sterol 14-α demethylase than fluconazole, with HDOCK scores of −207.97 (± 11.86) and −206.62 (± 11.51) for compounds **3a** and **7a**, respectively, compared to fluconazole’s score of −193.30 (± 11.13). These results suggest that compounds **3a** and **7a** may exert their antifungal activity through a similar, if not enhanced, mechanism to fluconazole, contributing to their efficacy against *Aspergillus* species. Interestingly, compounds **3a** and **7a** share several interface residues within their complex structures with the antifungal drug VT-1598 [61]. Specifically, compound **3a** shares the following residues with VT-1598: TYR 68, TYR 122, LEU 125, THR 126, TYR 136, PHE 234, ALA 307, GLY 308, SER 311, ILE 373, HIS 374, SER 375, LEU 503, and PHE 504. On the other hand, compound **7a** shares the following residues with VT-1598: TYR 68, TYR 122, PHE 234, ILE 373, HIS 374, SER 375, LEU 503, and PHE 504. Remarkably, these residues have been shown to play important roles in the antifungal activity of VT-1598. Notably, HIS 374 (acting as a proton donor) stands out as a critical residue for antifungal–protein binding, further corroborating its importance in the interaction between the compound and the protein target [61]. While fluconazole generally demonstrated superior effectiveness across *Alternaria alternata* 8126, *Ulocladium botrytis* 12027, and *Aureobasidium pullulans* 8269, which are notable for their roles in various research studies on fungal diseases [72,73], the presence of inhibitory effects from the synthesized compounds suggests for *Aspergillus* species potential for their use in combination therapies or as adjuncts to other drugs. These findings encourage further investigation into the mechanisms of action and structure–activity relationships of the synthesized compounds, aimed at optimization of their antifungal properties and development of new therapeutic strategies against fungal infections.

## 5. Conclusions

This study demonstrates the successful synthesis of novel Fmoc-protected 1,2,4-triazolyl-α-amino acids and their corresponding dipeptides, targeting antifungal activity against *Aspergillus* species, which are notorious pathogens in both human and veterinary medicine. The synthesized compounds were tested for their ability to inhibit the growth of multiple fungal strains, including *Aspergillus versicolor* and *Aspergillus flavus*. The solution-phase synthesis of the Fmoc-protected 1,2,4-triazolyl-α-amino acids **3a** and **3b**, followed by their conversion into the dipeptides **7a** and **7b**, involved an optimized strategy that included solvent selection, protection strategies, and the careful tuning of reaction conditions. Notably, a solvent system comprising 1,4-dioxane and acetone proved to be the most effective for protecting the triazole-containing amino acids, ensuring high yields and minimal side reactions. The development of a novel purification method to separate excess reagents, such as 9-fluorenylmethoxycarbonyl-N-oxysuccinimide ester, was an innovative contribution from a synthetic perspective. On the other hand, our antifungal activity studies revealed that the dipeptide **7a** exhibited superior antifungal effects to the antifungal drug fluconazole against *Aspergillus versicolor* and *Aspergillus flavus*. Our mechanistic studies based on molecular docking revealed that compounds **3a** and **7a** bind to a similar region of the sterol 14-α demethylase as fluconazole. The dose-dependent enhancement of the antifungal effect in the dipeptide **7a** suggests its potential as a more effective therapeutic agent. Although fluconazole was more effective against strains different from *Aspergillus*, some of the tested compounds still demonstrated detectable inhibitory effects, supporting the hypothesis that these new derivatives could serve as adjunctive or alternative therapies to conventional antifungals. The promising activity of the synthesized dipeptide **7a**, particularly against *Aspergillus* species, suggests that these structures could be valuable candidates for further development into treatments for fungal infections. Moreover, given the rising concern about antifungal resistance [74], these compounds could also serve as a platform for the design of next-generation antifungal agents that are both potent and less prone to resistance. In conclusion, the synthesis and characterization of Fmoc-protected 1,2,4-triazolyl-α-amino acids and dipeptides represents a significant step toward the development of new antifungal agents. The results of this study underscore the potential of these compounds in treating fungal infections, and their use could be an important strategy to mitigate the impact of *Aspergillus* infections in both humans and animals. Further research, including in vivo studies, is essential to fully assess the clinical applicability, toxicity profiles, and broader spectrum of activity of these compounds.

## Figures and Tables

**Table 1:** *Aspergillus* infections and their clinical manifestations in different animal species.

Species	*Aspergillus*-Related Conditions	Clinical Manifestations	Affected Systems/Organs
Birds	Aspergillosis	Respiratory distress, weight loss, lethargy	Primarily lungs (respiratory system)
Cattle	Mycotic abortion	Abortion or stillbirth, particularly in late pregnancy	Uterus, placenta
Horses	Laryngeal pouch mycosis	Respiratory issues, coughing, nasal discharge	Laryngeal pouch, respiratory system
	Mycotic keratitis	Eye infections, corneal ulcers, vision impairment	Eyes (cornea)
Dogs	Nasal/paranasal aspergillosis	Nasal discharge, facial pain, sinus issues	Nasal and paranasal tissues
	Intervertebral space infection	Neurological symptoms (e.g., ataxia, paralysis)	Spine (intervertebral spaces)
	Renal aspergillosis	Kidney dysfunction, potential kidney failure	Kidneys
Cats	Sinonasal, sino-orbital aspergillosis	Respiratory distress, nasal discharge, eye problems (e.g., conjunctivitis, exophthalmos)	Sinuses, orbital region, lungs (pulmonary system)

**Table 2:** Antifungal activity of test compounds against various fungal strains.

Concentration of Test Compounds		Names and Numbers of Strains According to the Microbial Depository Center					
		*Aspergillus versicolor* 12134	*A.flavus* 10567	*A.candidus* 10711	*Alternaria alternata* 8126	*Ulocladium botrytis* 12027	*Aureobasidium pullulans* 8269
Fluconazole	89 μM	+	+	-	++	++	+++
	183 μM	+	++	+	++	+++	+++
	278 μM	++	++	+	+++	+++	+++
**2a**	89 μM	-	-	-	-	-	-
	183 μM	+	-	-	+	-	+
	278 μM	+	+	-	+	+	+
**3a**	89 μM	++	+	-	-	+	-
	183 μM	++	+	+	+	+	+
	278 μM	++	++	++	++	++	++
**7a**	89 μM	+	+	+	+	+	-
	183 μM	++	++	+	++	++	-
	278 μM	++	++	++	++	++	-
**2b**	89 μM	-	-	-	-	-	-
	183 μM	+	+	-	-	-	-
	278 μM	+	+	+	+	+	-
**3b**	89 μM	+	+	-	+	+	-
	183 μM	+	+	+	+	+	+
	278 μM	-	++	+	++	++	++
**7b**	89 μM	-	+	-	-	-	-
	183 μM	-	+	+	-	-	-
	278 μM	+	++	+	+	+	-

“-”: No activity (inhibition % 0–10%); “+”: Low activity (inhibition % 10–30%); “++”: Moderate activity (inhibition % 40–70%); “+++”: High activity (inhibition % 70–90%).

**Table 3:** IC_50_ values and inhibition percentages of fluconazole, compound **3a**, and compound **7a** at a concentration of 555 μM against *Aspergillus* species.

*Aspergillus* Species	Compound	Inhibition at 555 μM (%)	IC_50_ (μM)	SD
*Aspergillus versicolor* 12134	fluconazole	95	254.01	0.05
	**3a**	90	-	0.09
	**7a**	97	169.94	0.09
*Aspergillus flavus* 10567	fluconazole	85	184.64	0.09
	**3a**	80	267.86	0.06
	**7a**	95	176.69	0.1
*Aspergillus candidus* 10711	fluconazole	85	476.20	0.08
	**3a**	70	240.35	0.04
	**7a**	95	248.94	0.04

**Table 4:** HDOCK scores for the top 1 pose and average scores for poses 1–3 (± standard deviation) of fluconazole and compounds **3a** and **7a** are presented. The receptor interface residues for the top 1 poses are listed with their respective ligand—residue distances in Angstroms (Å). Common residues across all complexes are also indicated.

Compound	HDOCK Score (Top 1 Pose)	HDOCK Score (Avg. Top 1–3 ± SD)	Receptor Interface Residues for the Top 1 Poses (Residue—Ligand Distance/Å)	Common Residues
Fluconazole	−193.30	−183.27 ± 11.13	TYR 122 (3.460 Å), LEU 125 (4.013 Å), THR 126 (3.484 Å), PHE 130 (3.176 Å), VAL 135 (3.295 Å), TYR 136 (3.261 Å), ALA 307 (2.987 Å), GLY 308 (4.884 Å), SER 311 (3.536 Å), ILE 373 (3.680 Å), LEU 503 (4.080 Å), PHE 504 (2.951 Å), HEM 580 (2.982 Å)	TYR 122, LEU 125, THR 126, TYR 136, ALA 307, GLY 308, SER 311, ILE 373, LEU 503, PHE 504, HEM 580
**3a**	−207.97	−192.63 ± 11.86	TYR 68 (2.876 Å), LEU 91 (3.496 Å), VAL 121 (3.717 Å), TYR 122 (1.493 Å), LEU 125 (2.678 Å), THR 126 (1.979 Å), TYR 136 (4.045 Å), PHE 229 (4.538 Å), PHE 234 (2.725 Å), ALA 307 (2.652 Å), GLY 308 (4.381 Å), SER 311 (3.302 Å), ILE 373 (2.803 Å), HIS 374 (3.010 Å), SER 375 (3.111 Å), ILE 376 (3.278 Å), ILE 377 (3.117 Å), ARG 378 (4.992 Å), ASN 398 (4.823 Å), TYR 500 (4.472 Å), SER 502 (4.805 Å), LEU 503 (2.285 Å), PHE 504 (3.955 Å), HEM 580 (3.095 Å)	TYR 122, LEU 125, THR 126, TYR 136, ALA 307, GLY 308, SER 311, ILE 373, LEU 503, PHE 504, HEM 580
**7a**	−206.62	−190.42 ± 11.51	THR 65 (3.017 Å), ILE 66 (3.865 Å), TYR 68 (3.957 Å), GLY 69 (2.937 Å), ILE 70 (3.821 Å), LEU 91 (2.091 Å), LEU 92 (3.069 Å), GLY 93 (3.464 Å), LYS 94 (2.725 Å), THR 96 (4.979 Å), TYR 122 (4.669 Å), PRO 231 (3.321 Å), ILE 232 (3.429 Å), PHE 234 (2.594 Å), MET 235 (2.112 Å), ILE 373 (4.311 Å), HIS 374 (3.240 Å), SER 375 (2.883 Å), ILE 377 (3.910 Å), TYR 500 (2.672 Å), SER 501 (3.521 Å), SER 502 (2.803 Å), LEU 503 (3.029 Å), PHE 504 (4.955 Å)	TYR 122, ILE 373, LEU 503, PHE 504

## Data Availability

The original contributions presented in the study are included in the article; further inquiries can be directed to the corresponding authors.

## Supplementary Materials

The following supporting information can be downloaded at: https://www.mdpi.com/article/10.3390/biom15010061/s1, including NMR, ESI MS, and IR characterization spectra, biological data related to antifungal assays, and in silico data. Figure S1. Photographs from the antifungal test (visual inspection) showing the antifungal activities of the compounds developed in the present work, alongside fluconazole as the reference antifungal drug, against the following fungal strains: *A. versicolor* 12134 (I), *A. flavus* 10567 (II), *A. candidus* 10711 (III), *Aureobasidium pullulans* 8269 (IV), *Alternaria altenata* 8126 (V), *Ulocladium botrytis* 12027 (VI); Figure S2. Plots of inhibition rates versus concentration for the determination of IC50 values (Table 3) for the following fungal strains: (a, b, c) *A. versicolor* 12134; (d, e, f) *A. flavus* 10567; and (g, h, i) *A. candidus* 10711; Figure S3. 3D interaction diagrams for the complexes of Comp 3a, Comp 7a, and fluconazole with the protein as obtained using PLIP (Protein-Ligand Interaction Profiler).
