# Supplementary material for: Synthesis and Antifungal Activity of Fmoc-Protected 1,2,4-Triazolyl-α-Amino Acids and Their Dipeptides Against Aspergillus Species
Source: Biomolecules. 2025 Jan 4;15(1):61. doi: 10.3390/biom15010061 (PMC11762334; doi:10.3390/biom15010061)
Supplement: Supplementary file 1 [file biomolecules-15-00061-s001.zip › biomolecules-3357339-supplementary.pdf]

Supporting Information

Synthesis Synthesis and Antifungal Activity of Fmoc-Protected 1,2,4-Triazolyl-α-Amino Acids and Their Dipeptides Against Aspergillus Species

Tatevik Sargsyan[1,2], Lala Stepanyan[1], Henrik Panosyan[3], Heghine Hakobyan[1], Monika Israyelyan[1], Avetis Tsaturyan[1,2], Nelli Hovhannisyan[1,2], Caterina Vicidomini[4], Anna Mkrtchyan[1,2], Ashot Saghyan[1,2*], Giovanni Roviello[4*]

[1] Scientific and Production Center "Armbiotechnology" NAS RA, 14 Gyurjyan Str., Yerevan, 0056, Armenia; armbiotech@gmail.com

[2] Institute of Pharmacy, Yerevan State University, 1 Alex Manoogian Str., Yerevan 0025, Armenia; info@ysu.am

[3] Scientific Technological Center of Organic and Pharmaceutical Chemistry 26, Azatutian ave., Yerevan, 0014 Republic of Armenia; stcopc {[at]} sci.am

[4] Institute of Biostructures and Bioimaging, Italian National Council for Research (IBB-CNR), Area di Ricerca Site and Headquarters, Via Pietro Castellino 111, 80131 Naples, Italy

NMR (1H and 13C NMR) spectra for all intermediates and final peptides are shown in this supporting information file.

1H NMR 9-fluorenylmethoxycarbonyl-(*S*)-β-4-allyl-3- (2-methoxyphenyl)-5-thioxo-1,2,4-triazol-1-yl]-alanine (3a) DMSO/CCl_4 1/3

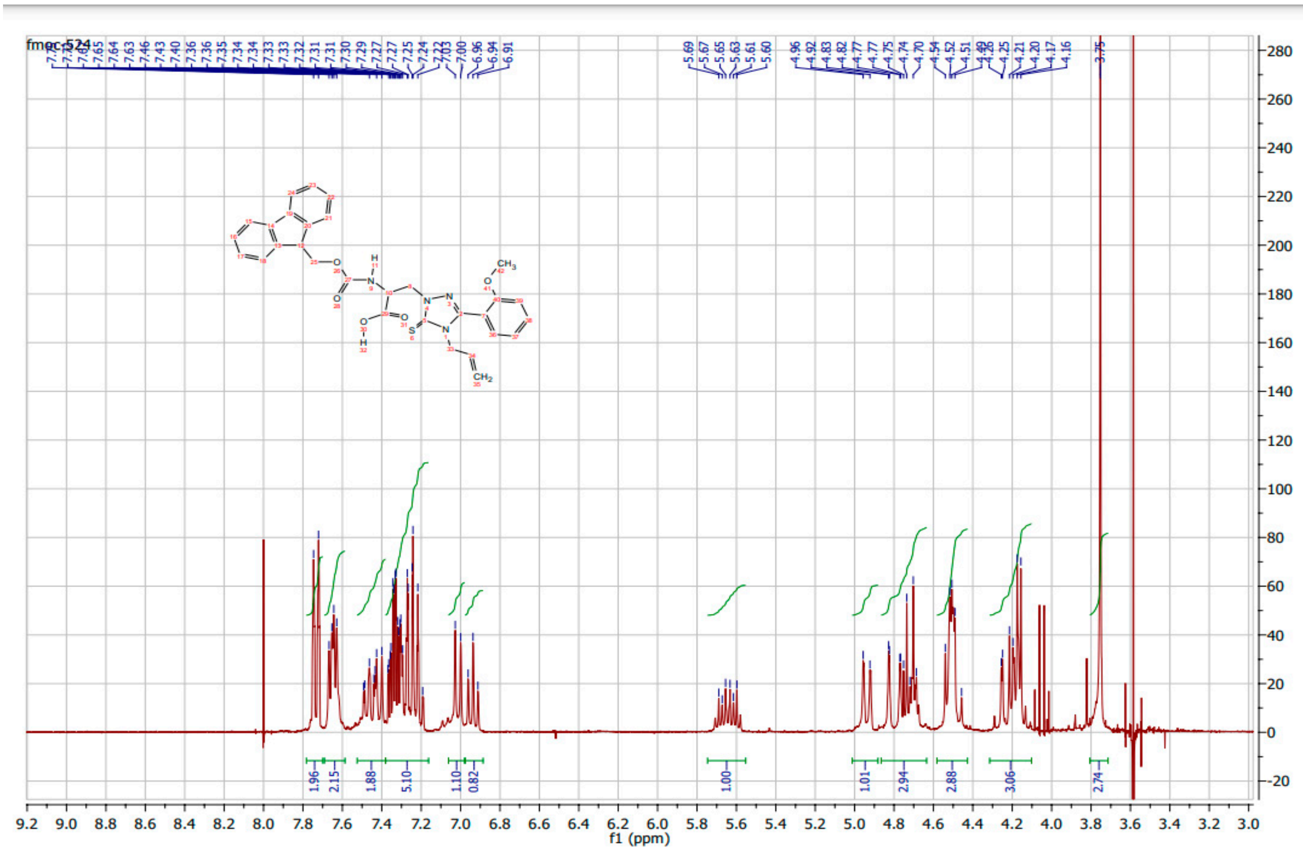

13C NMR 9-fluorenylmethoxycarbonyl-(*S*)-β-4-allyl-3- (2-methoxyphenyl)-5-thioxo-1,2,4- triazol-1-yl]-alanine (3a) DMSO/CCl_4 1/3

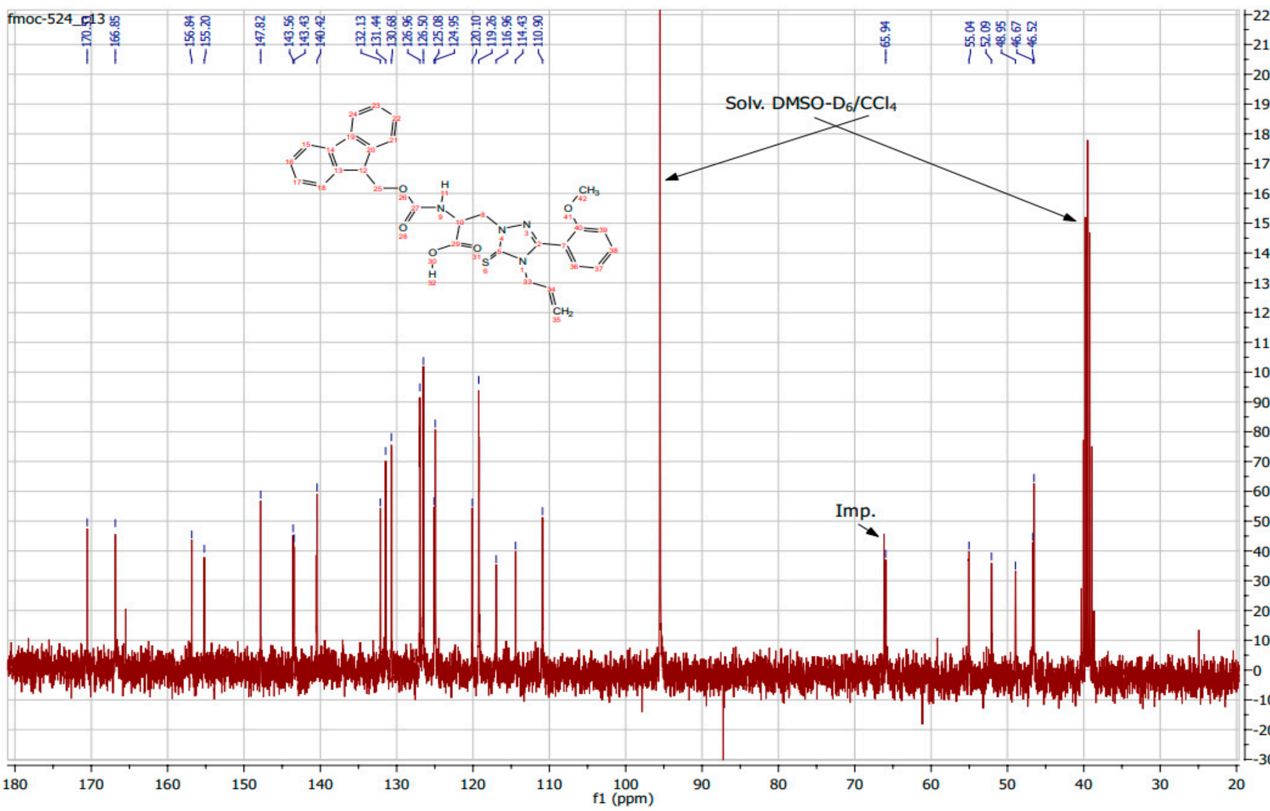

1H NMR 9-fluorenylmethoxycarbonyl-(*S*)-β-[4-allyl-3-(furan-2-yl)- 5-thioxo-1,2,4-triazol-1-yl]-alanine (3b) DMSO/CCl_4 1/3

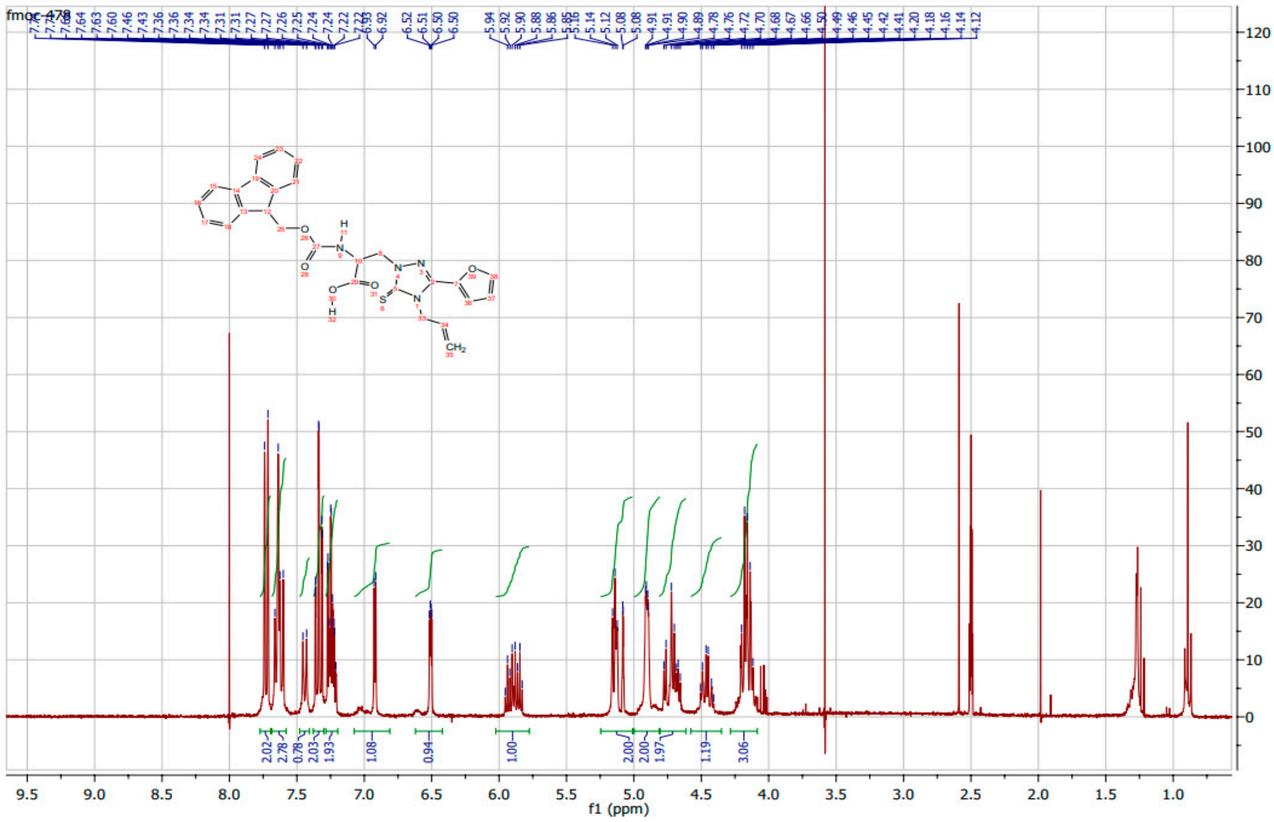

13C NMR 9-fluorenylmethoxycarbonyl-(*S*)-β-[4-allyl-3-(furan-2-yl)- 5-thioxo-1,2,4-triazol-1-yl]-alanine (3b) DMSO/CCl_4 1/3

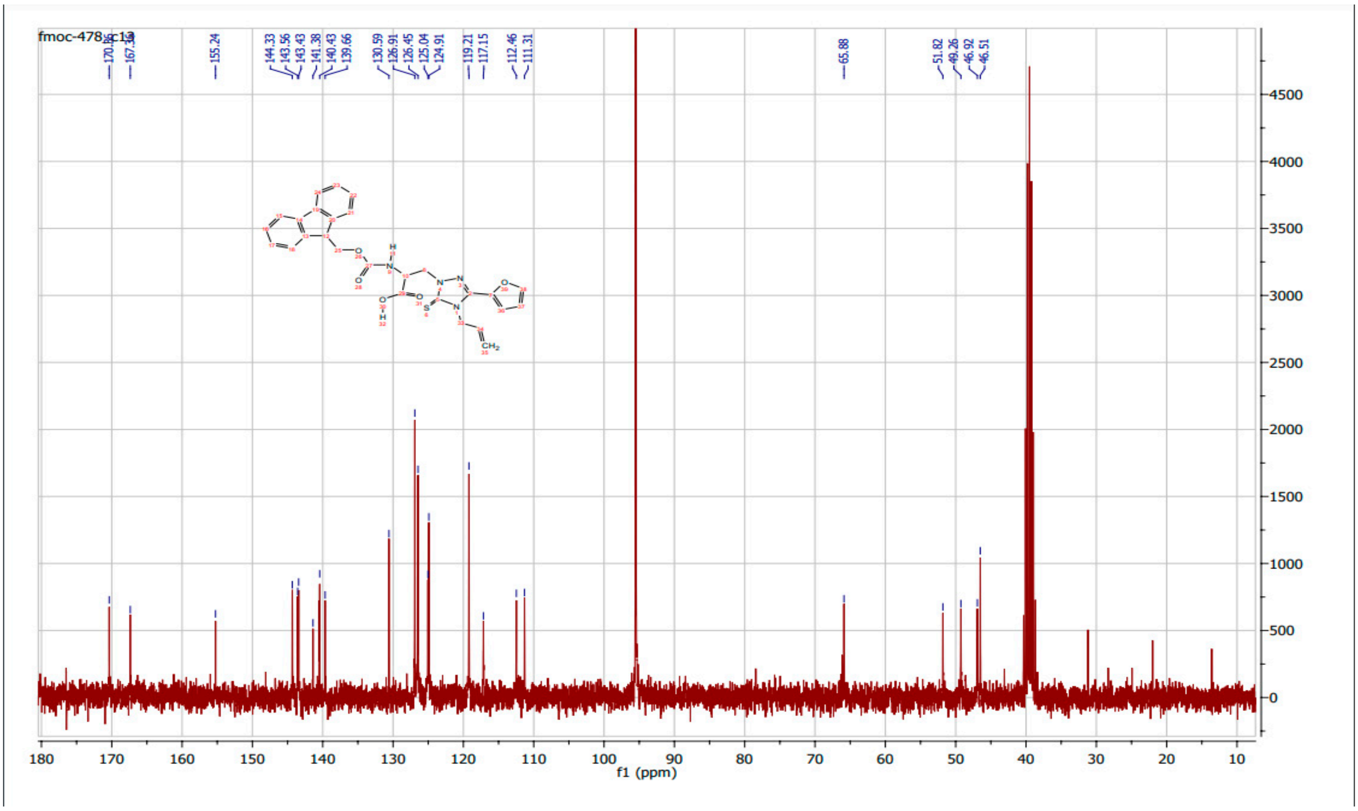

1H NMR 9-fluorenylmethoxycarbonyl-(*S*)-β-[4-allyl-3- (2-methoxyphenyl)-5-thioxo-1,2,4–triazol -1-yl]-*α*-alanylglycine (7a)

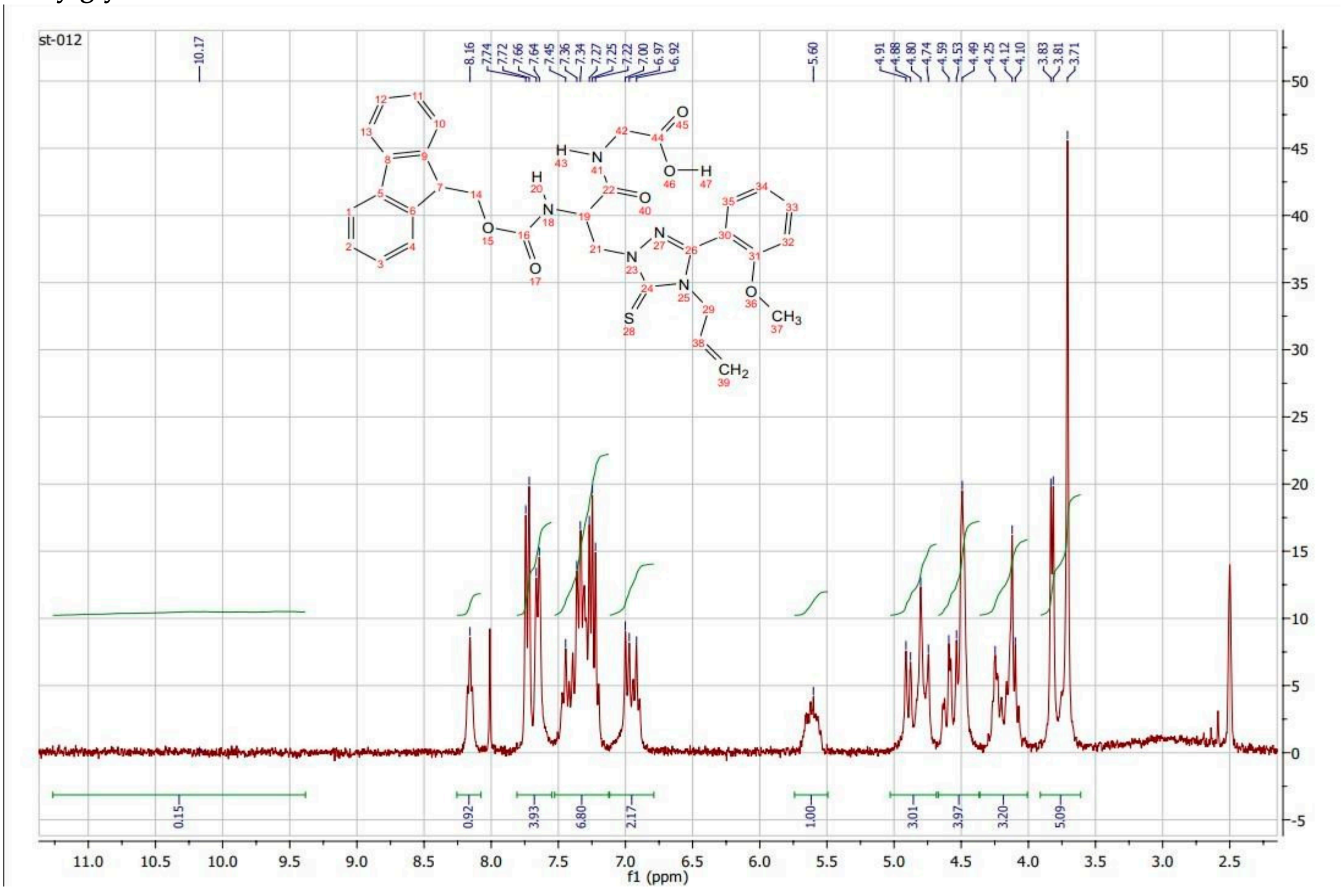

13C NMR 9-fluorenylmethoxycarbonyl-(*S*)-β-[4-allyl-3- (2-methoxyphenyl)-5-thioxo-1,2,4–triazol -1-yl]-α-alanylglycine (7a)

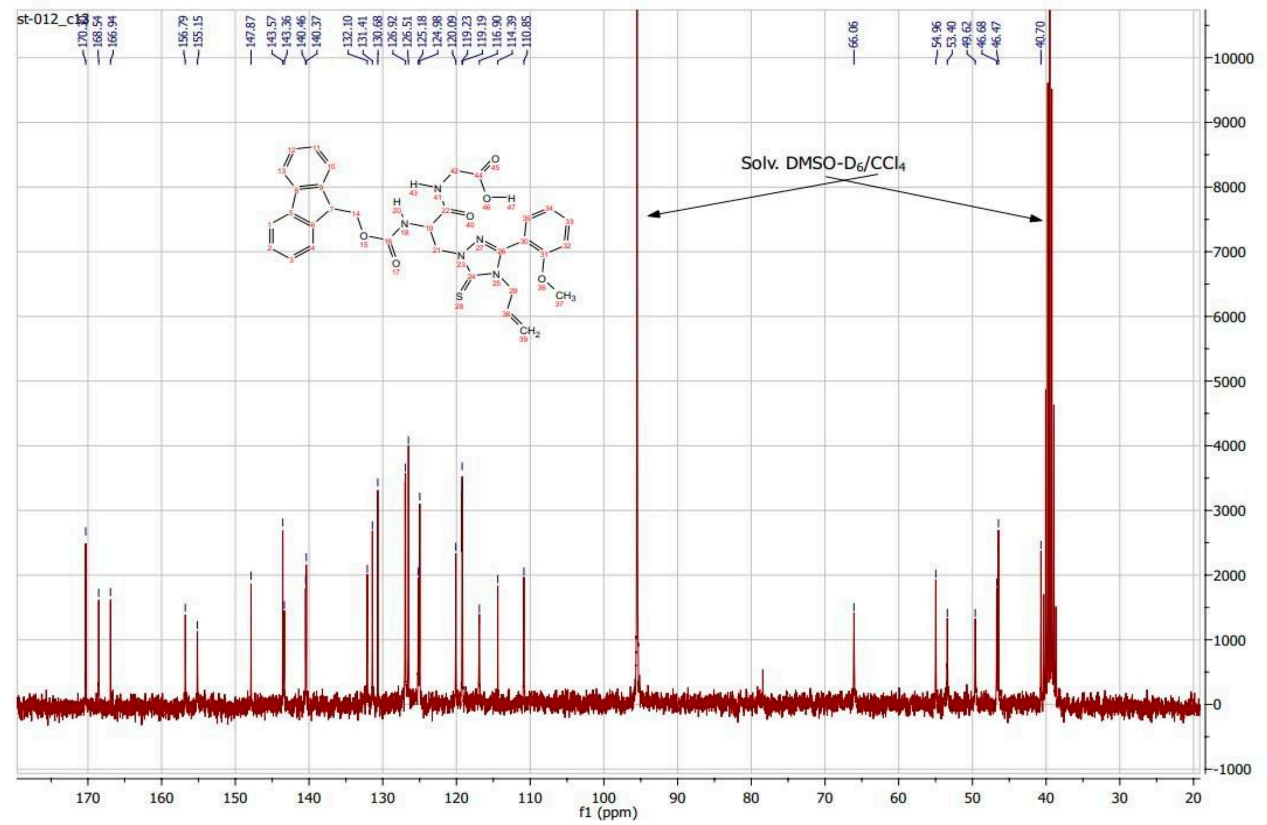

1H NMR 9-fluorenylmethoxycarbonyl-(*S*)-β-[4-allyl-3-(furan-2-yl)-5-thioxo-1,2,4-triazol-1-yl]-α-alanylglycine (7b) DMSO/CCl₄ 1/3

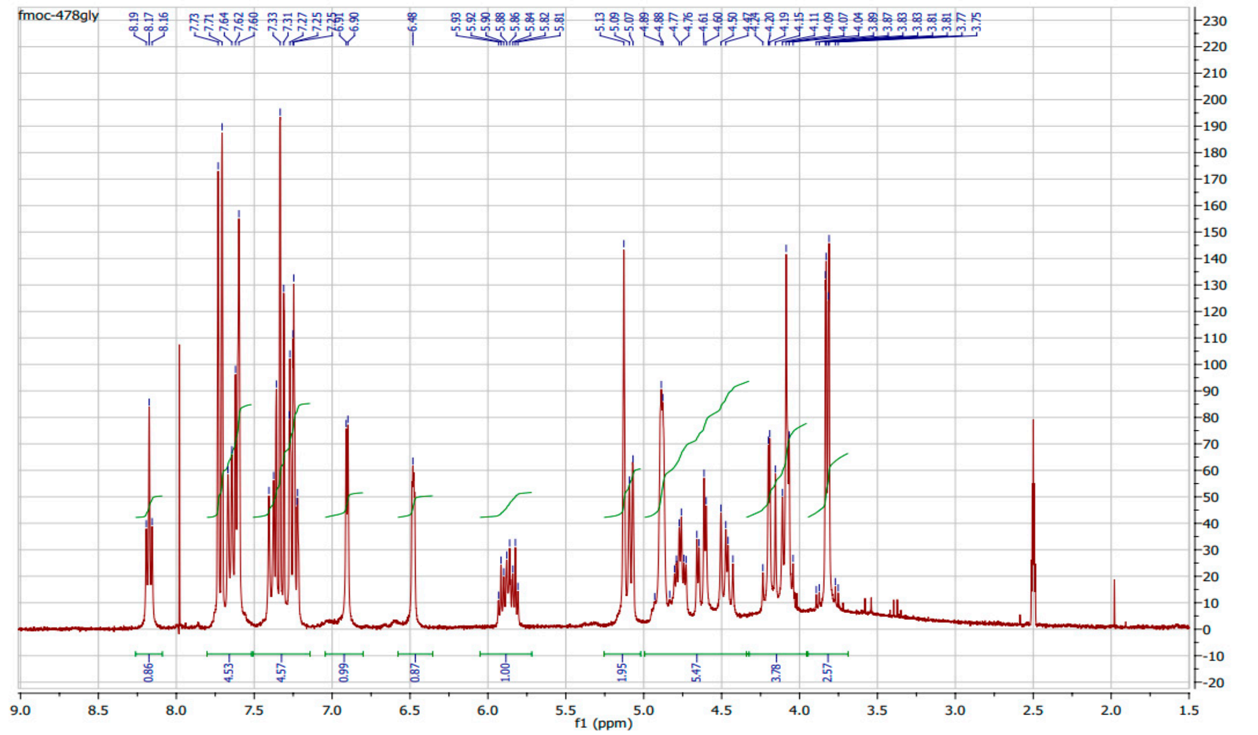

13C NMR 9-fluorenylmethoxycarbonyl-(*S*)-β-[4-allyl-3-(furan-2-yl)-5-thioxo-1,2,4-triazol-1-yl]-*α*-alanylglycine (7b) DMSO/CCl_4 1/3

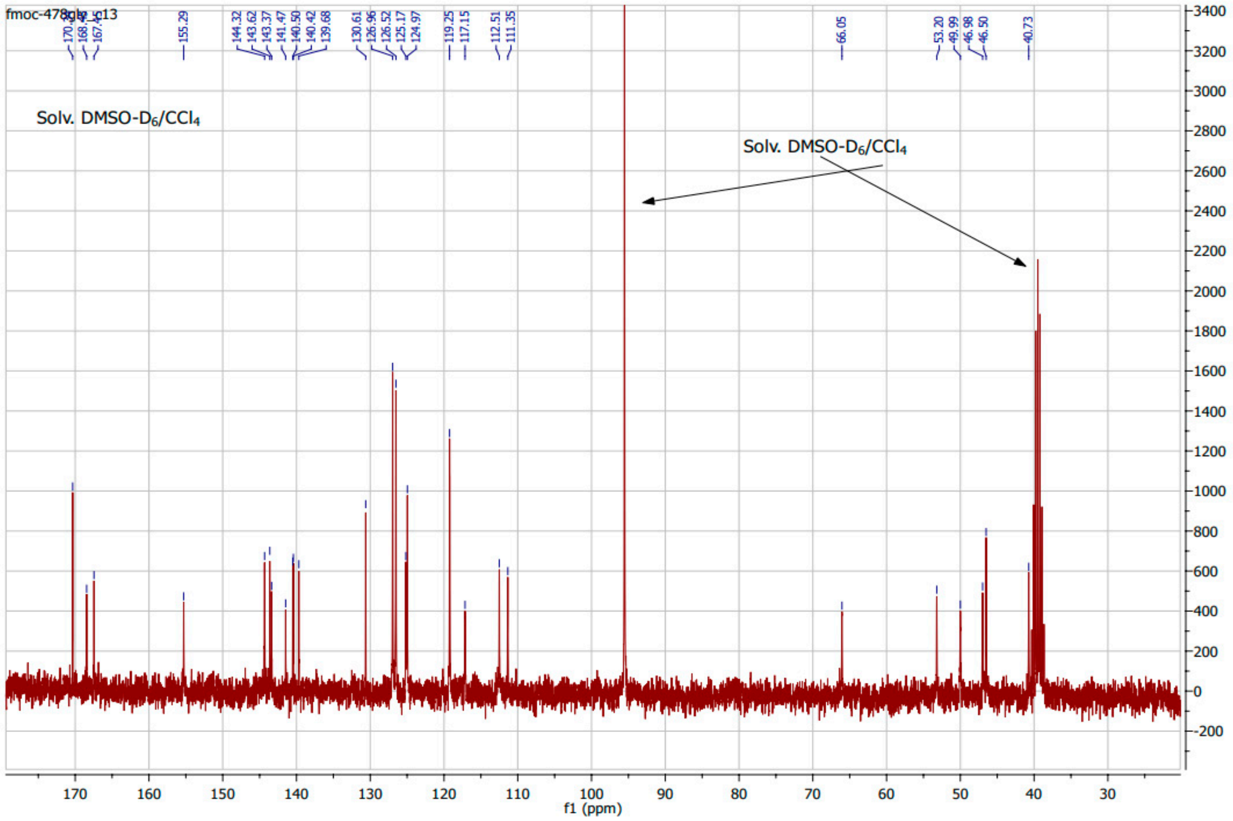

ESI MS: Sample Name: **9-fluorenylmethoxycarbonyl-(*S*)-β-4-allyl-3- (2-methoxyphenyl)-5-thioxo-1,2,4-triazol-1- yl]-alanine (3a)**

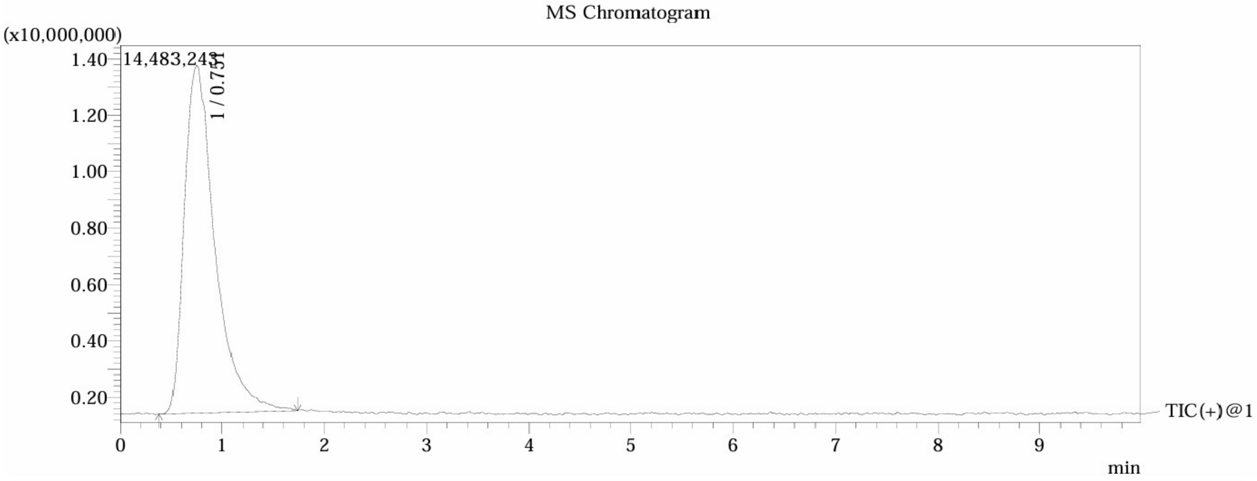

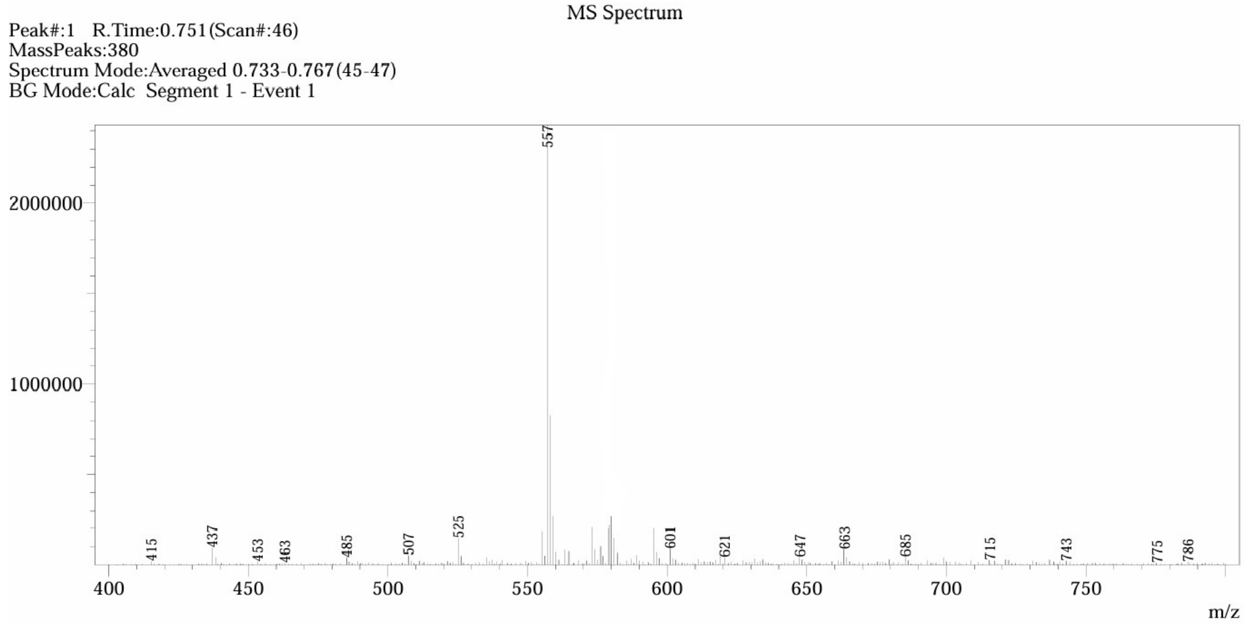

Peak #	Ret. Time	Area	Base Peak m/z
1	0.751	260246271	557.10

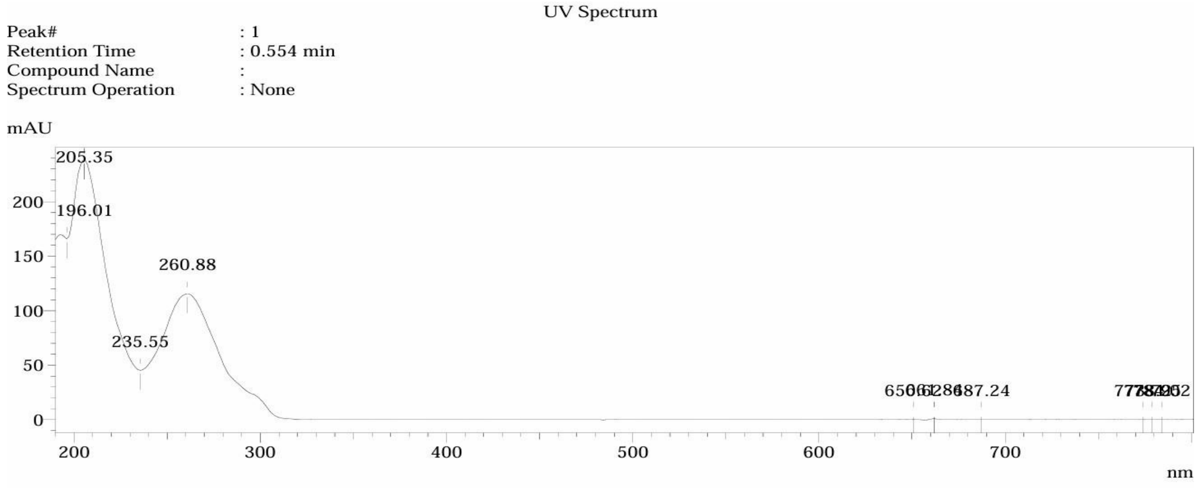

ESI MS: Sample Name: **9-fluorenylmethoxycarbonyl-(*S*)-β-[4-allyl-3-(furan-2-yl)-5-thioxo-1,2,4-triazol-1-yl]-alanine (3b)**

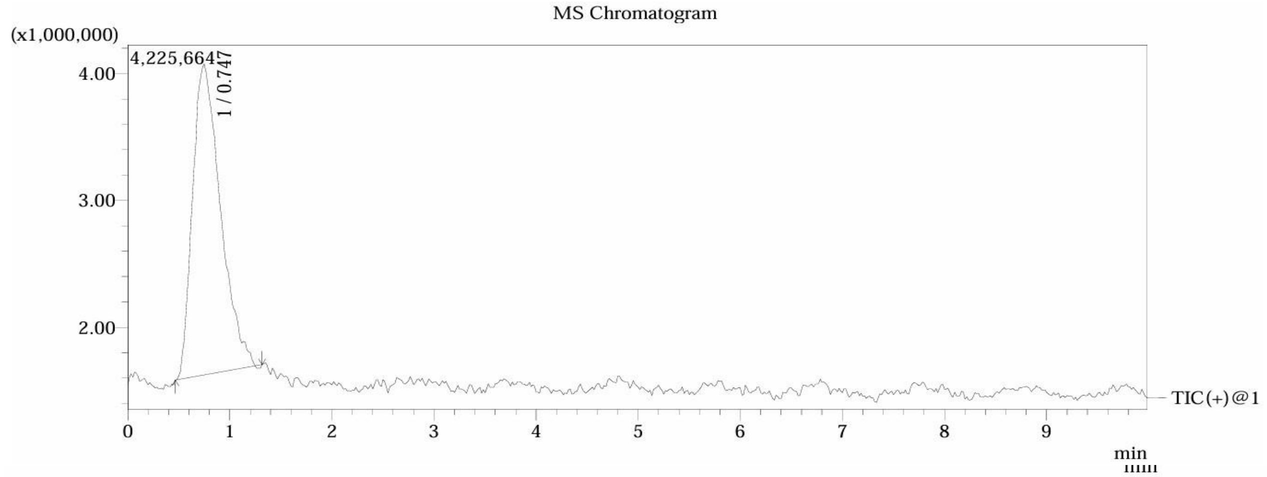

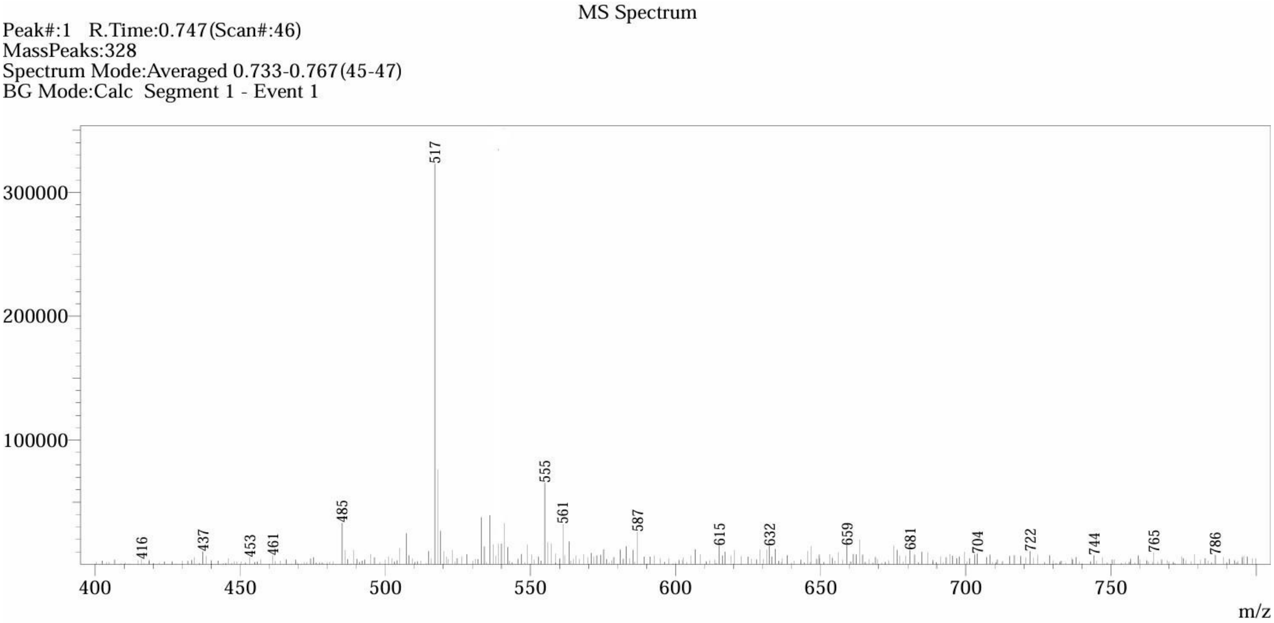

Peak #	Ret. Time	Area	Base Peak m/z
1	0.747	46667675	517.00

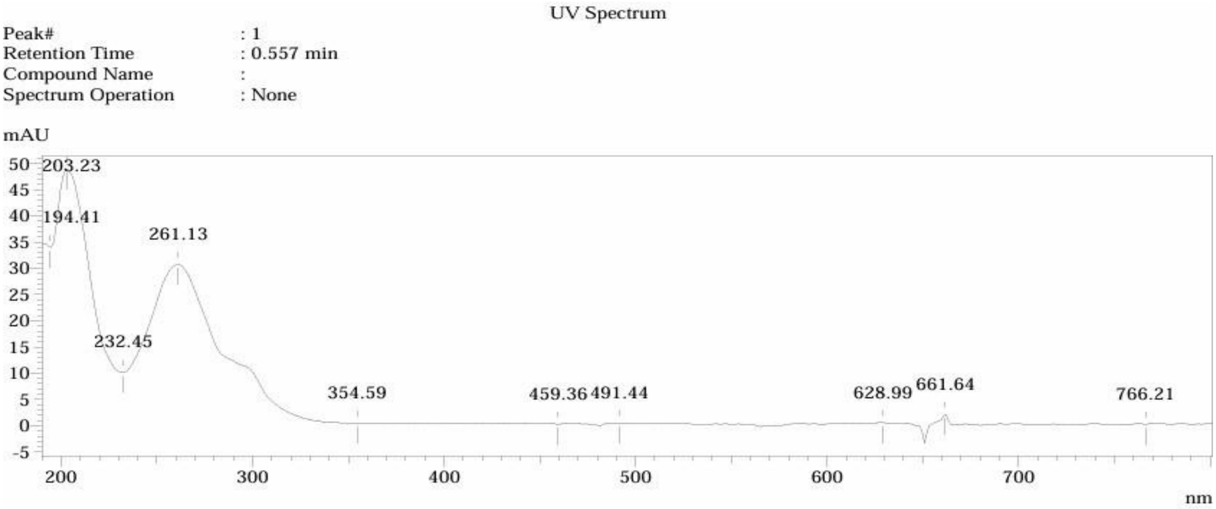

ESI MS: Sample Name: **9-fluorenylmethoxycarbonyl-(*S*)-β-[4-allyl-3- (2-methoxyphenyl)-5-thioxo-1,2,4–triazol -1- yl]-α-alanylglycine (7a)**

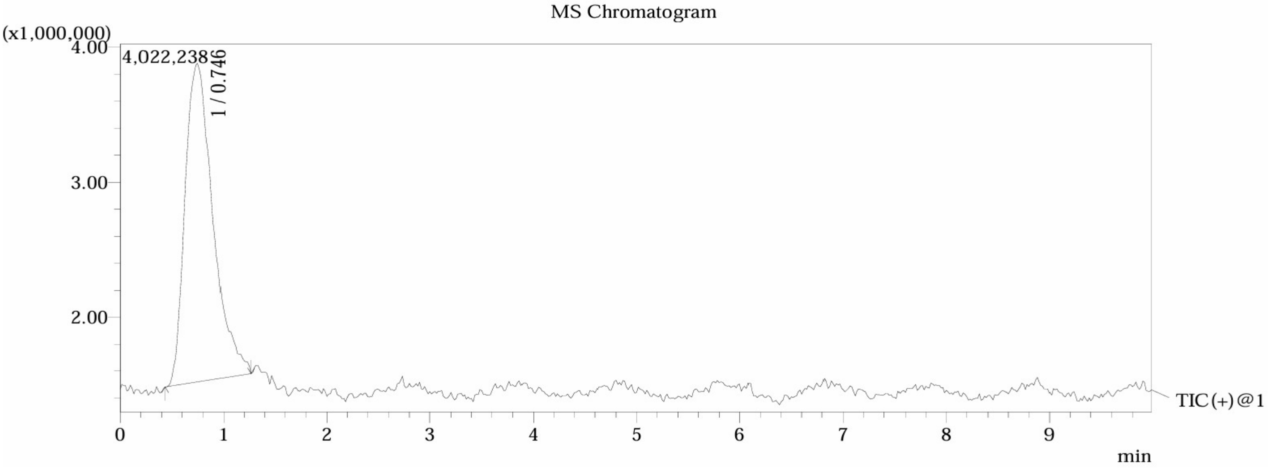

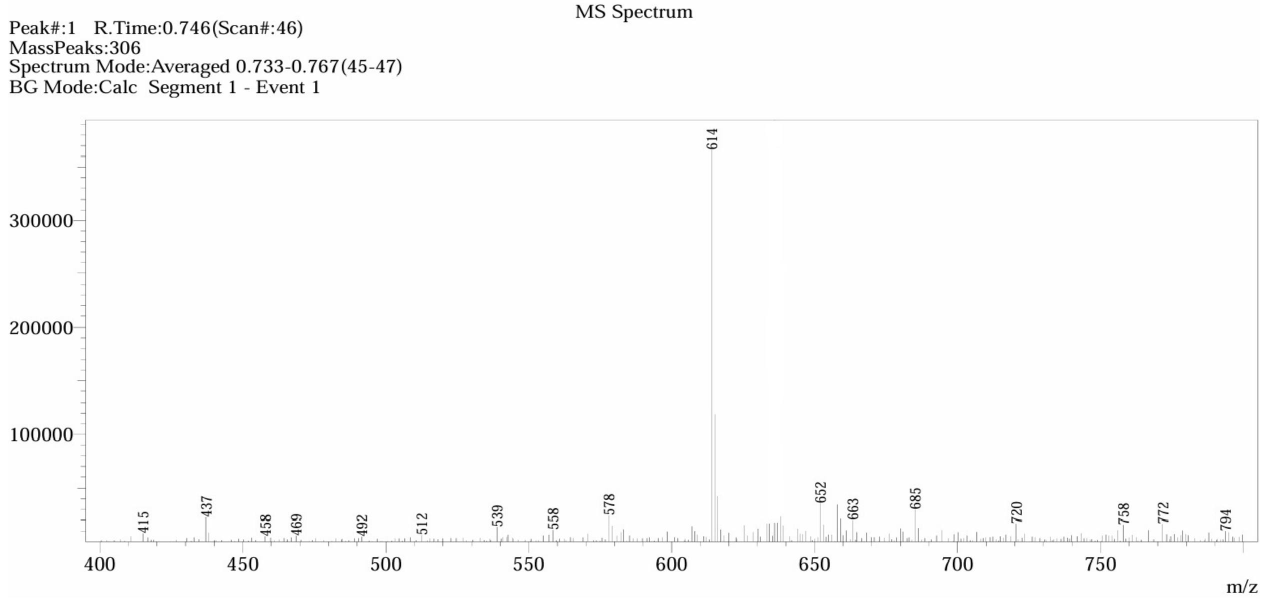

Peak #	Ret. Time	Area	Base Peak m/z
1	0.746	44774013	614.01

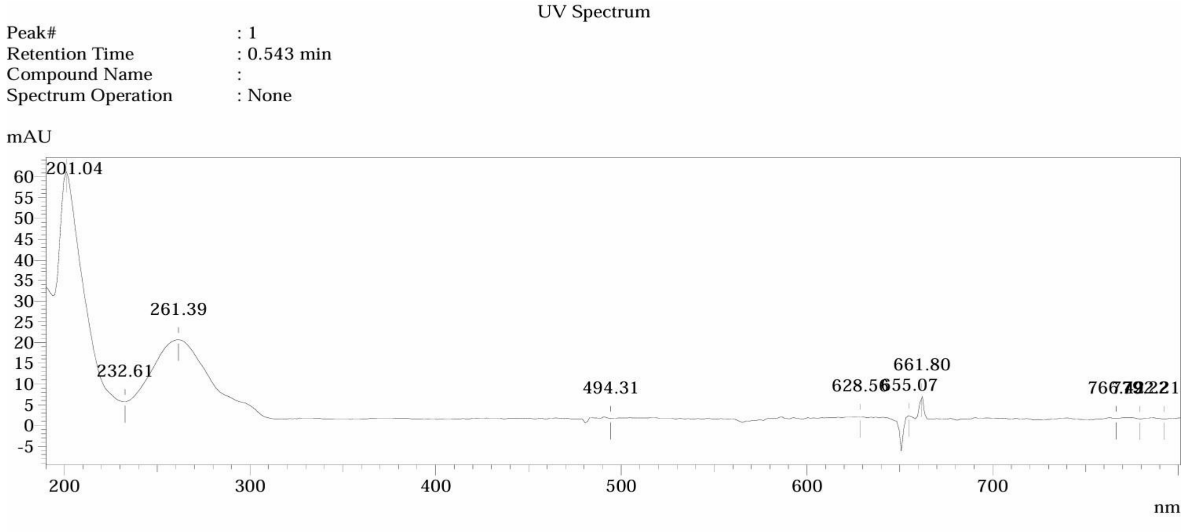

ESI MS: Sample Name: **9-fluorenylmethoxycarbonyl-(*S*)-β-[4-allyl-3-(furan-2-yl)-5-thioxo-1,2,4-triazol-1-yl]-*α*- alanylglycine (7b)**

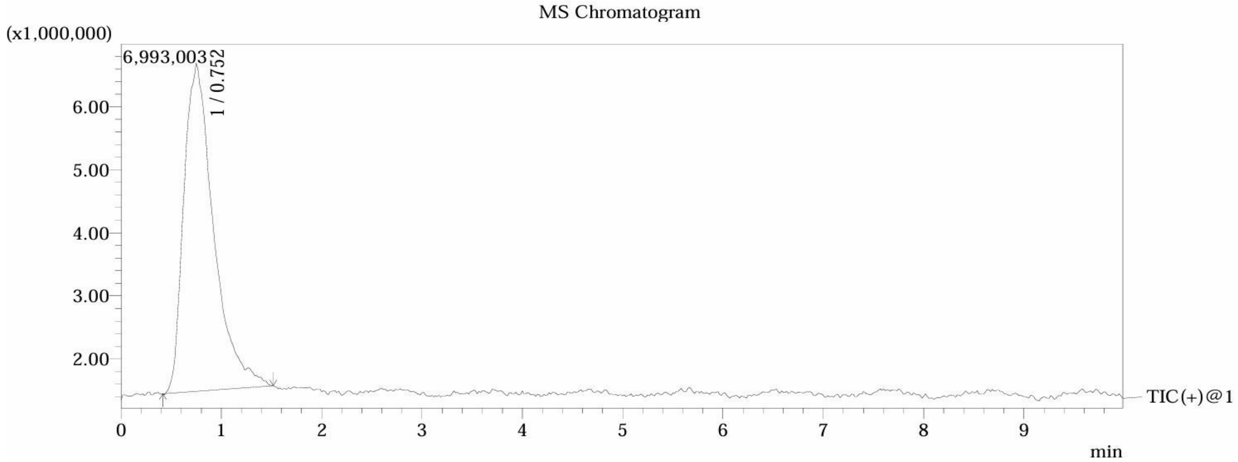

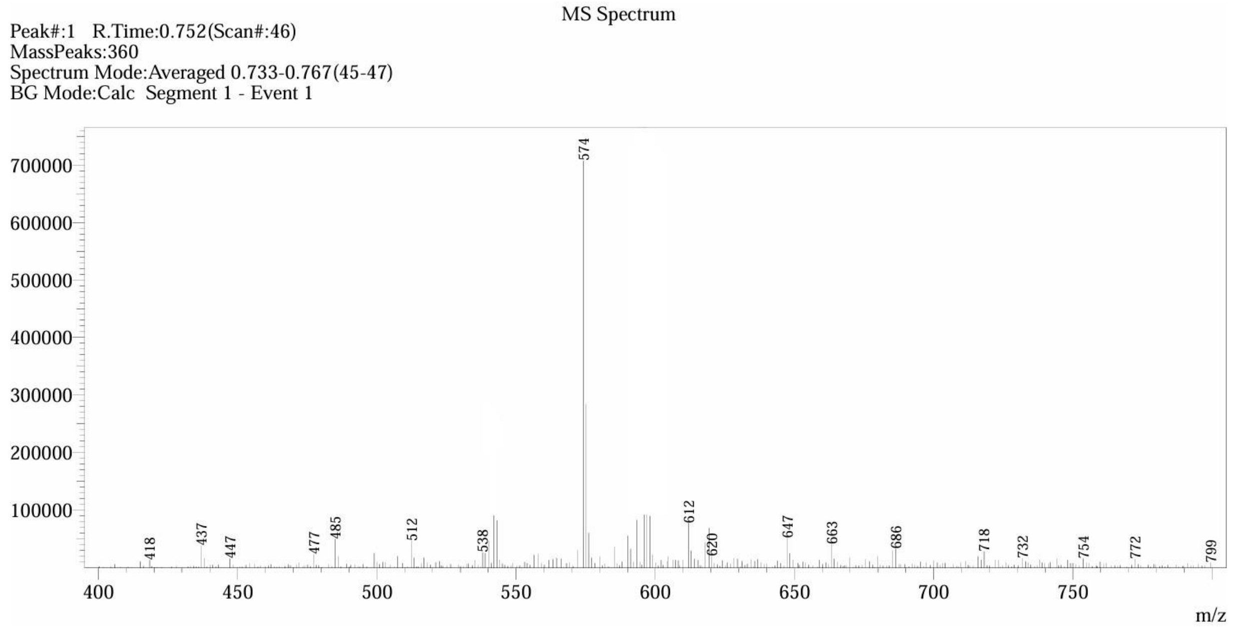

Peak #	Ret. Time	Area	Base Peak m/z
1	0.752	107695407	574.05

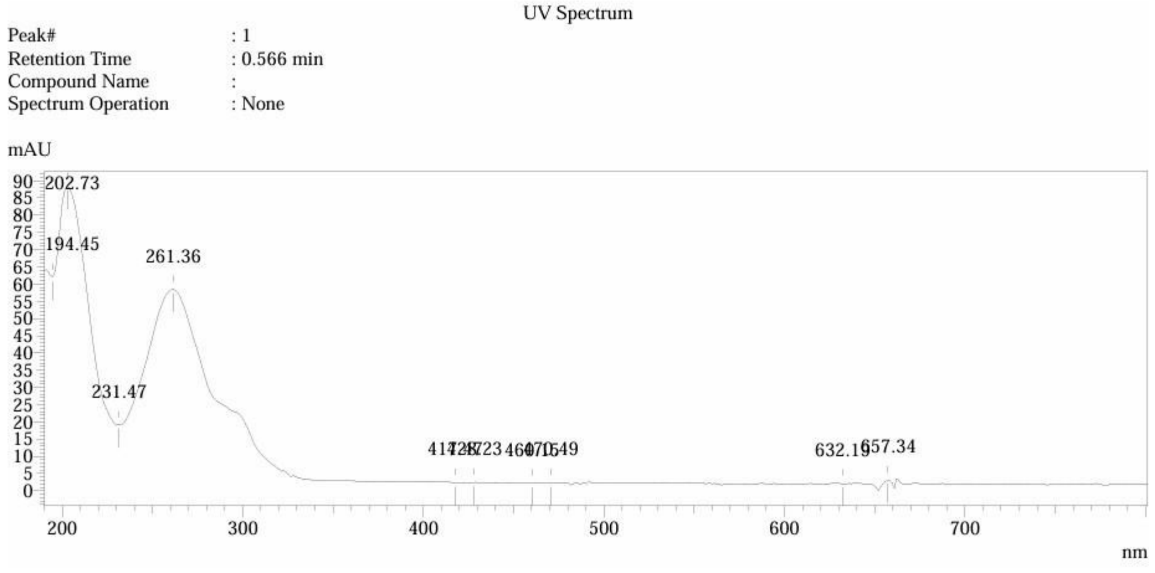

ATR-IR, ν, cm−1: 9-fluorenylmethoxycarbonyl-(*S*)-β-4-allyl-3- (2-methoxyphenyl)-5-thioxo-1,2,4-triazol-1-yl]-alanine (3a)

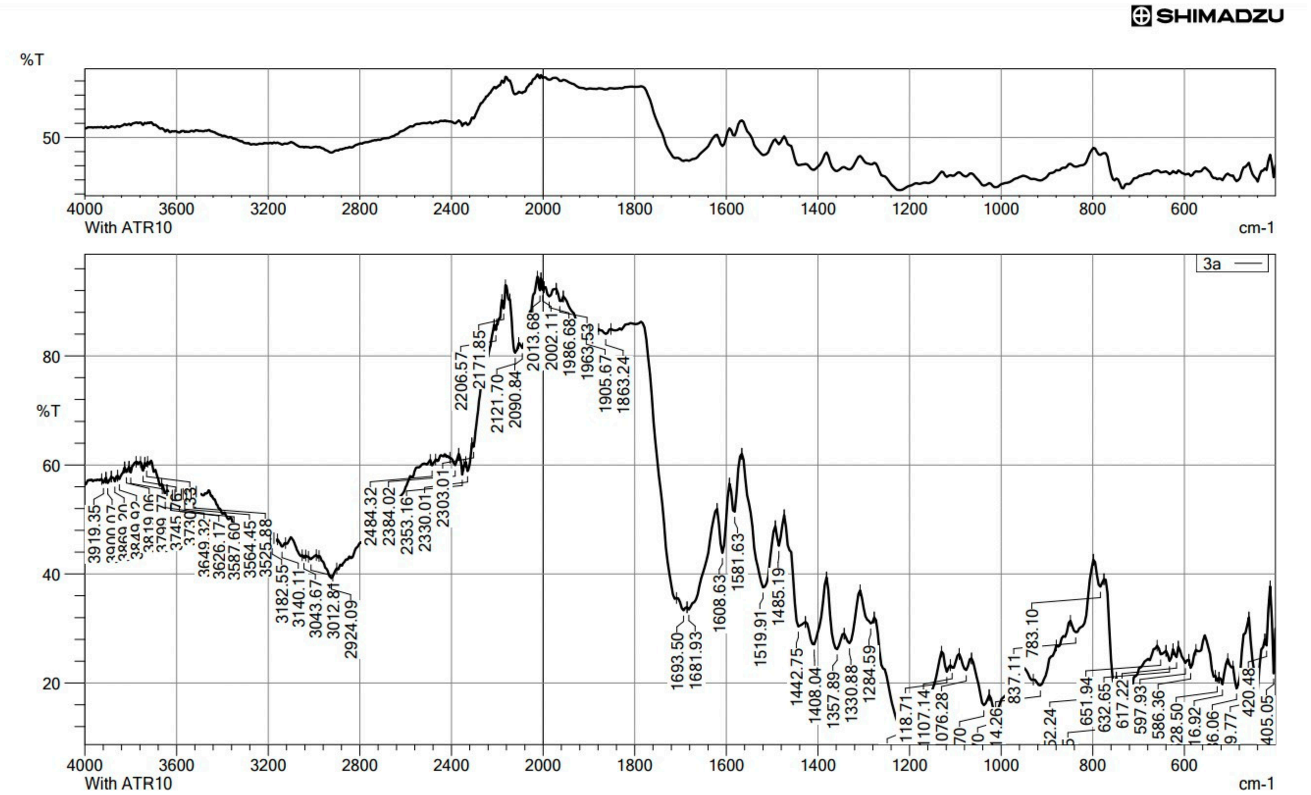

ATR-IR, ν, cm−1: 9-fluorenylmethoxycarbonyl-(*S*)-β-[4-allyl-3-(furan-2-yl)-5-thioxo-1,2,4-triazol-1-yl]-alanine (3b)

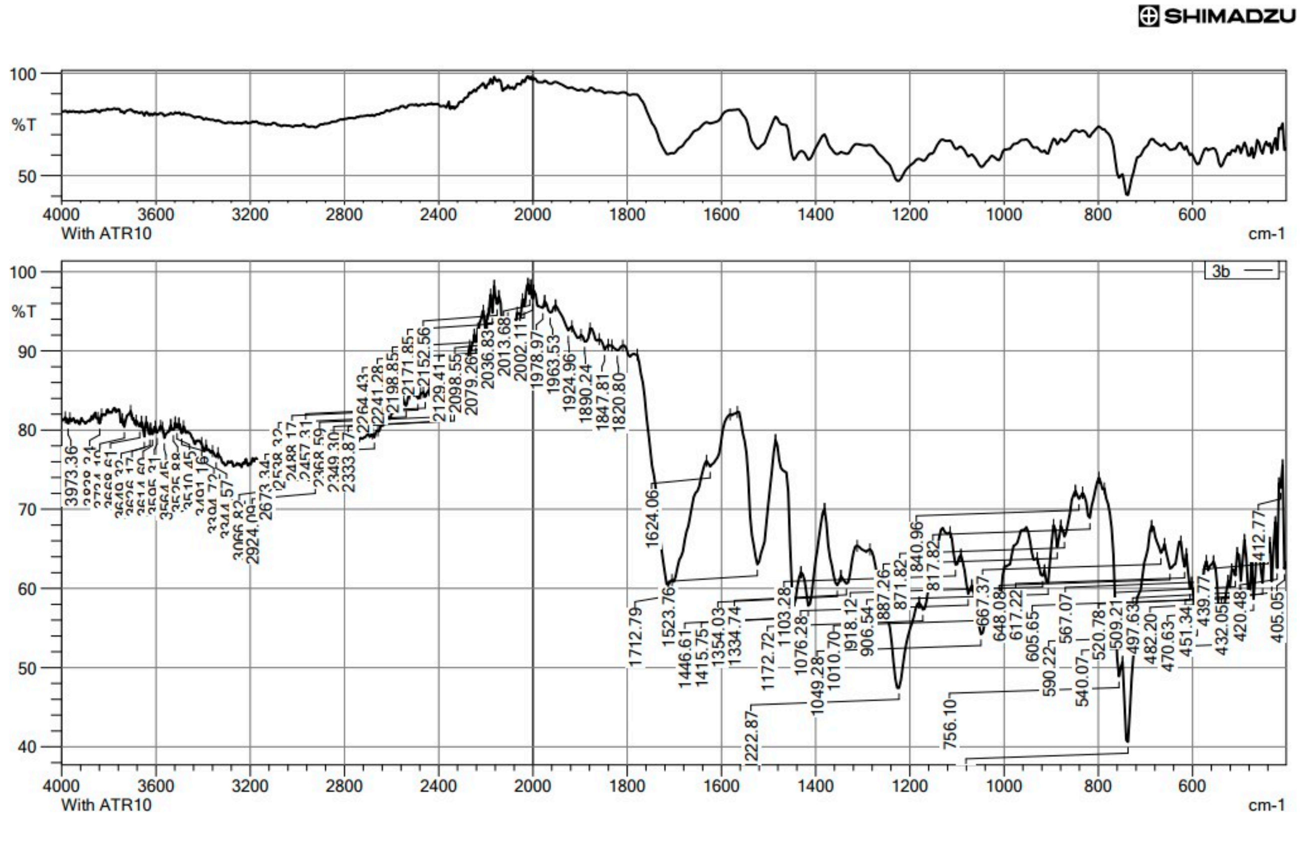

9-fluorenylmethoxycarbonyl-(*S*)-β-[4-allyl-3- (2-methoxyphenyl)-5-thioxo-1,2,4–triazol -1- yl]-*α*-alanylglycine (7a)

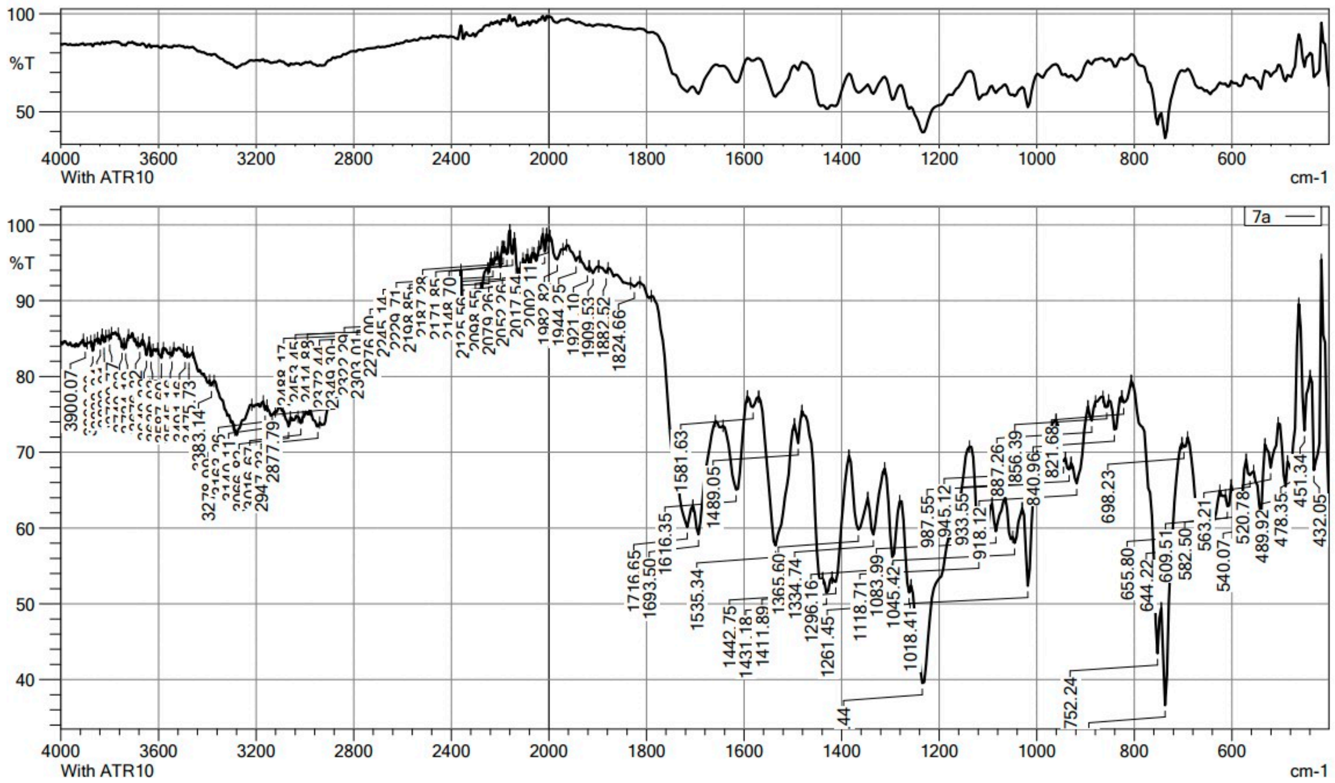

9-fluorenylmethoxycarbonyl-(*S*)-β-[4-allyl-3-(furan-2-yl)-5-thioxo-1,2,4-triazol-1-yl]-*α*- alanylglycine (7b)

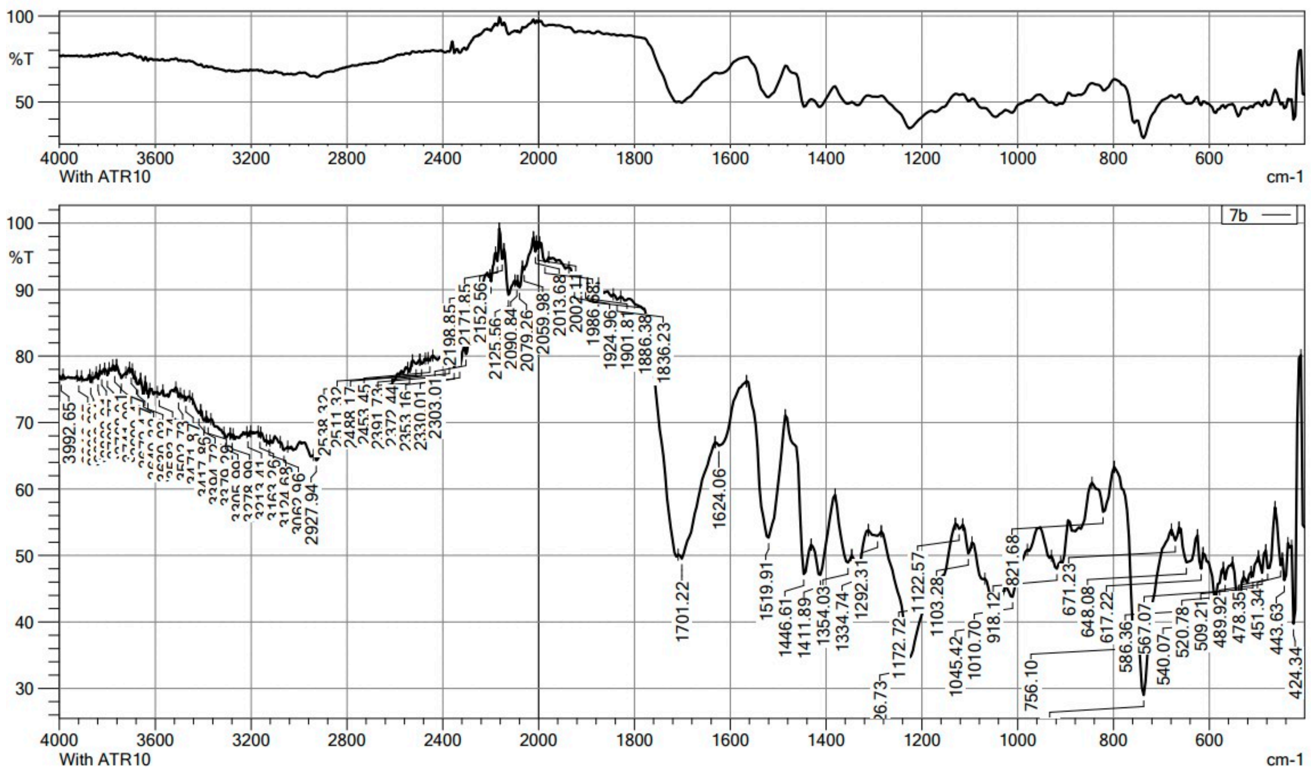

Antifungal activity: Photographs from the antifungal test (visual inspection) showing the antifungal activities of the compounds developed in the present work, alongside fluconazole as the reference antifungal drug, against the following fungal strains:
A. versicolor **12134 (I),** ***A. flavus*** **10567 (II),** ***A. candidus*** **10711 (III),** ***Aureobasidium pullulans*** **8269 (IV),** ***Alternaria altenata*** **8126 (V),** ***Ulocladium botrytis*** **12027 (VI).**

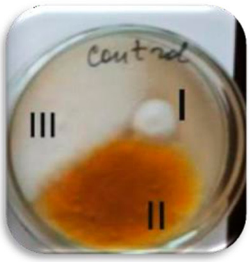

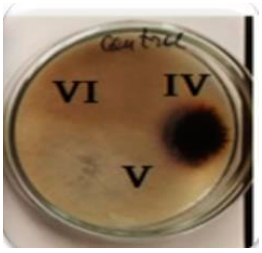

Compounds	278 µM 183 µM 89 µM 444 µM 555 µM	278 µM 183 µM 89 µM
Fluconazole	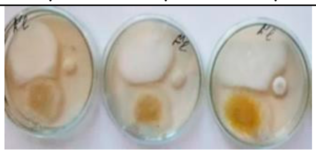 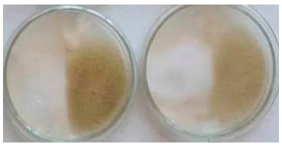	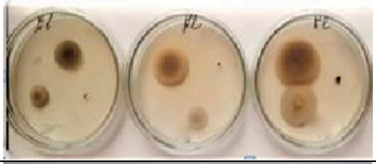
**2a**	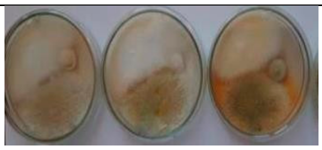	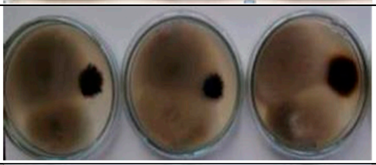
**3a**	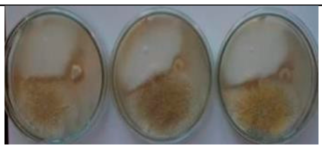 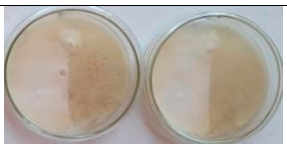	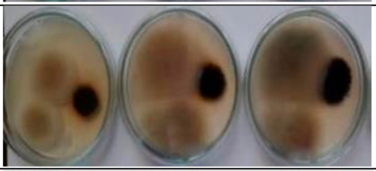
**7a**	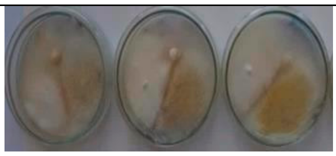 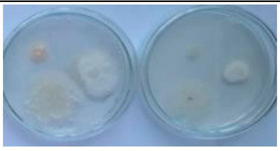	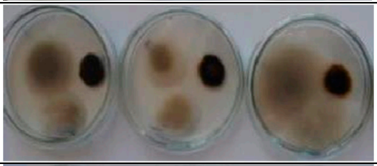
**2b**	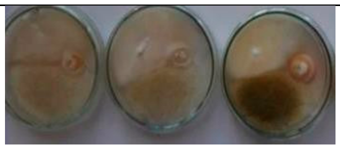	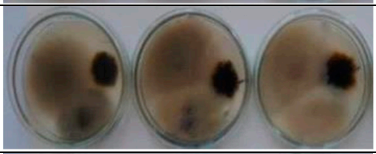
**3b**	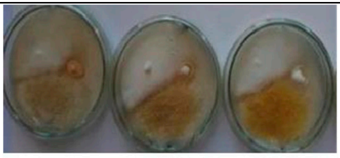	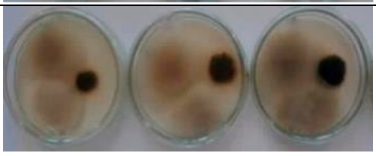
**7b**	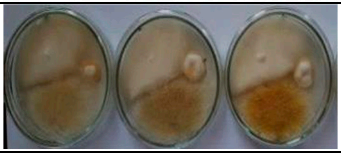	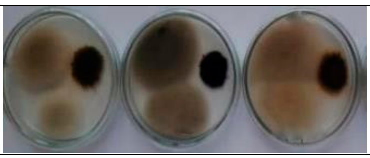

Figure S1. Photographs from the antifungal test (visual inspection) showing the antifungal activities of the compounds developed in the present work, alongside fluconazole as the reference antifungal drug, against the following fungal strains: *A. versicolor* 12134 (I), *A. flavus* 10567 (II), *A. candidus* 10711 (III), *Aureobasidium pullulans* 8269 (IV), *Alternaria altenata* 8126 (V), *Ulocladium botrytis* 12027 (VI). These images illustrate the inhibitory effects observed at different concentrations of the compounds, compared to the standard fluconazole treatment.

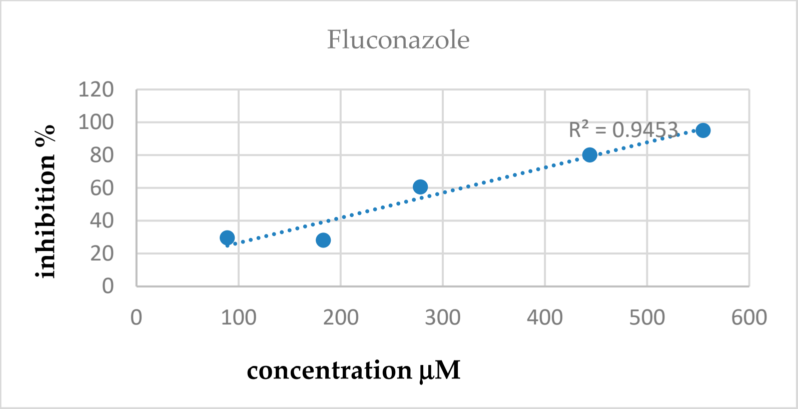
Fluconazole
120
100
80
60
40
20
0
0
100
200
300
400
500
600
R² = 0.9453
inhibition %
concentration µM

a

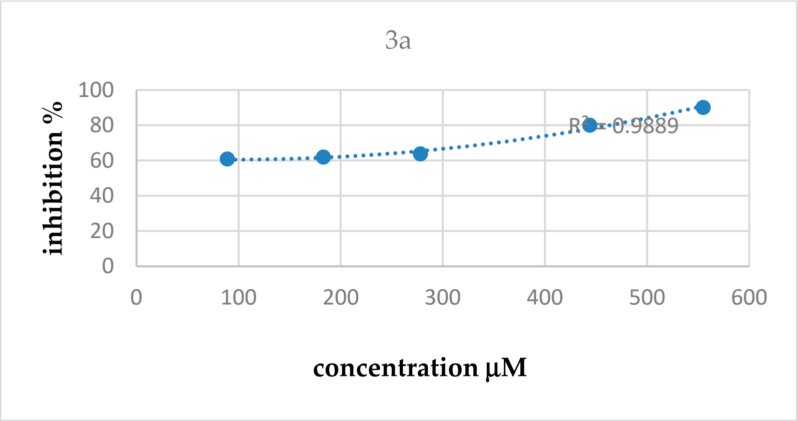
3a
100
80
60
40
20
0
0
100
200
300
400
500
600
R² = 0.9889
inhibition %
concentration µM

b

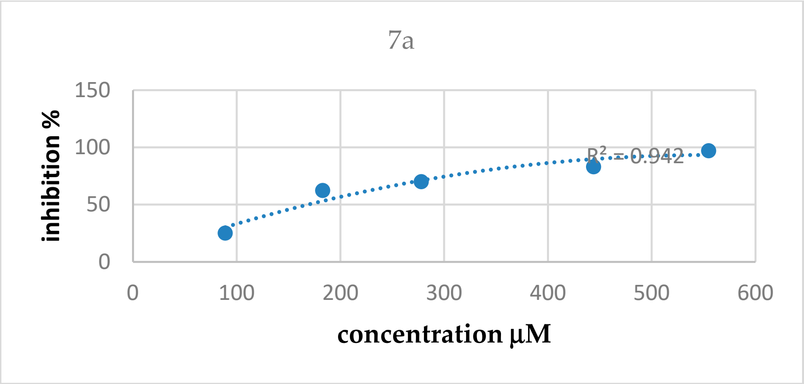
7a
150
100
50
0
0
100
200
300
400
500
600
R² = 0.942
inhibition %
concentration µM

c

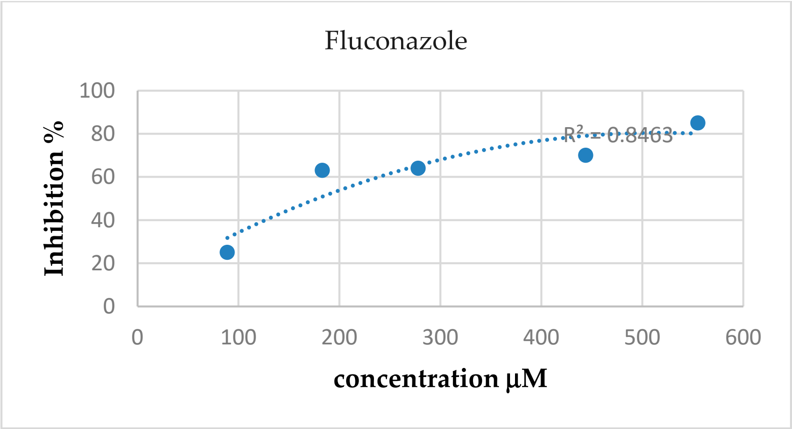
Fluconazole
100
80
60
40
20
0
0
100
200
300
400
500
600
R² = 0.8463
Inhibition %
concentration µM

d

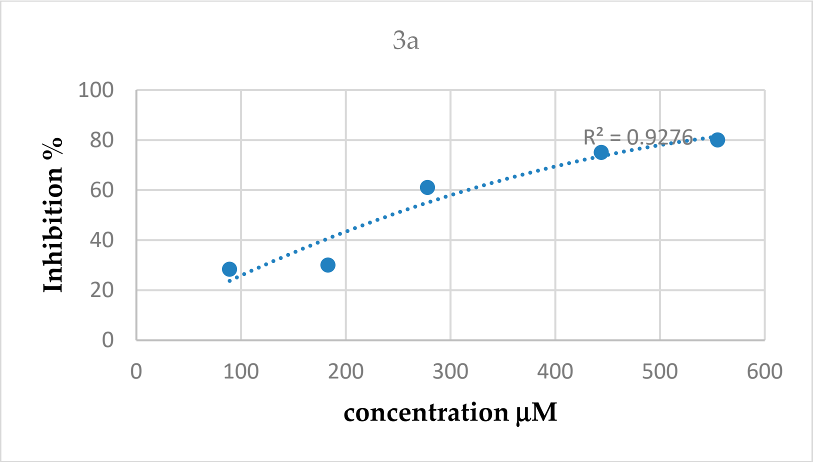
3a
100
80
60
40
20
0
0
100
200
300
400
500
600
Inhibition %
concentration µM
R² = 0.9276

e

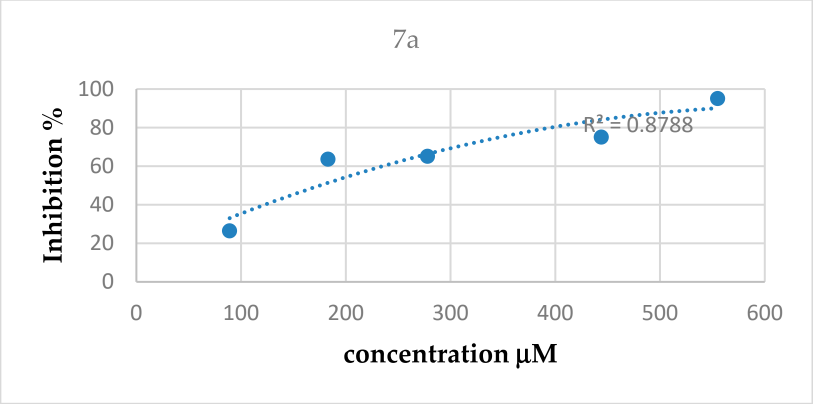
7a
100
80
60
40
20
0
0
100
200
300
400
500
600
Inhibition %
concentration µM
R² = 0.8788

f

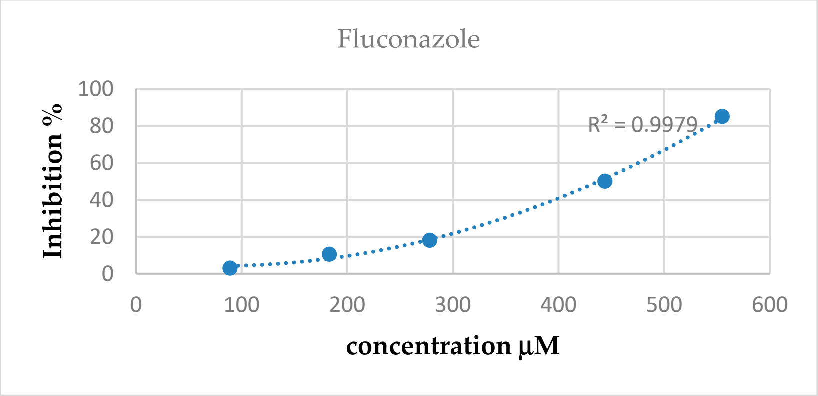
Fluconazole
100
80
60
40
20
0
0
100
200
300
400
500
600
Inhibition %
concentration µM
R² = 0.9979

g

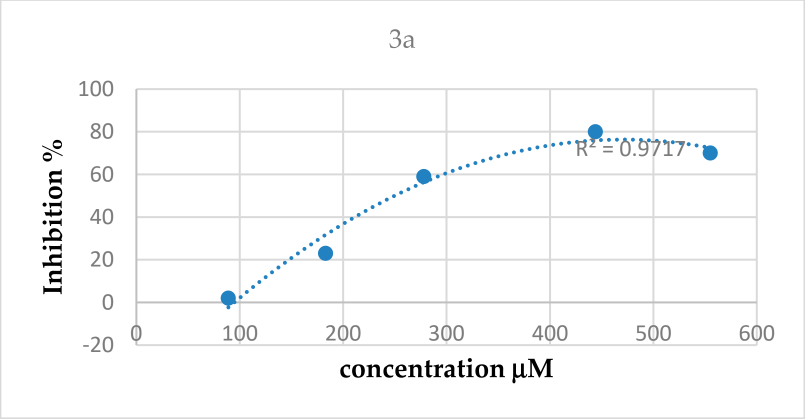
3a
100
80
60
40
20
0
-20
0
100
200
300
400
500
600
Inhibition %
concentration µM
R² = 0.9717

h

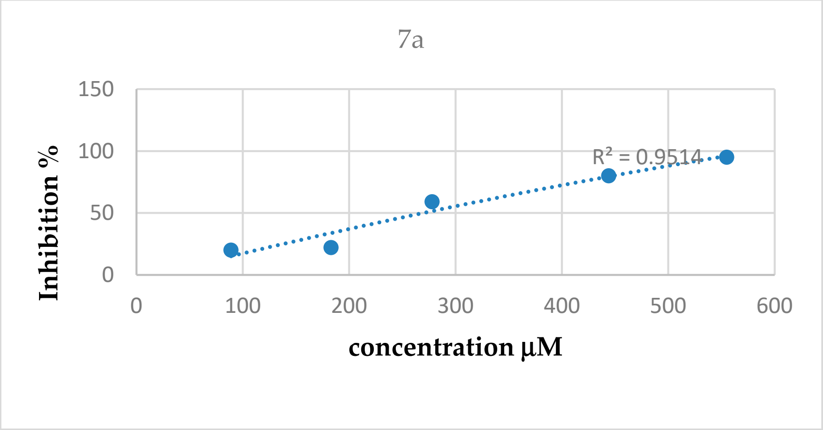

i

Figure S2. Plots of inhibition rates versus concentration for the determination of IC_{50} values (Table 3) for the following fungal strains: (a, b, c) *A. versicolor* 12134; (d, e, f) *A. flavus* 10567; and (g, h, i) *A. candidus* 10711.

Compound 3a – protein complex

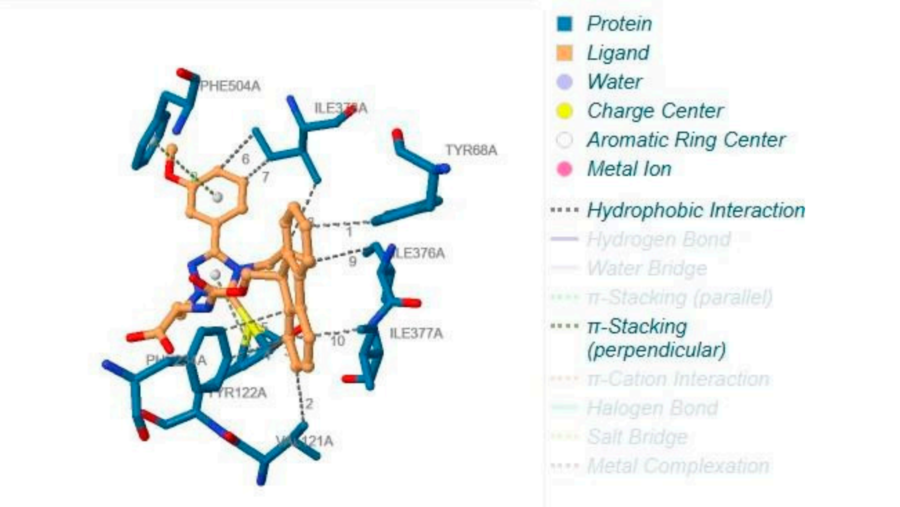

Hydrophobic Interactions ····

Index	Residue	AA	Distance	Ligand Atom	Protein Atom
1	68A	TYR	2.88	3825	157
2	121A	VAL	3.72	3831	584
3	122A	TYR	3.98	3830	594
4	234A	PHE	2.73	3830	1485
5	234A	PHE	3.04	3829	1484
6	373A	ILE	3.35	3800	2598
7	373A	ILE	2.80	3801	2596
8	373A	ILE	3.59	3807	2597
9	376A	ILE	3.46	3808	2620
10	377A	ILE	3.12	3830	2630

π-Stacking ···· ····

Index	Residue	AA	Distance	Angle	Offset	Stacking Type	Ligand Atoms
1	122A	TYR	4.31	71.82	1.48	T	3804, 3805, 3809, 3811, 3835
2	504A	PHE	5.35	72.71	1.91	T	3799, 3800, 3801, 3802, 3803, 3836

Compound 7a – protein complex

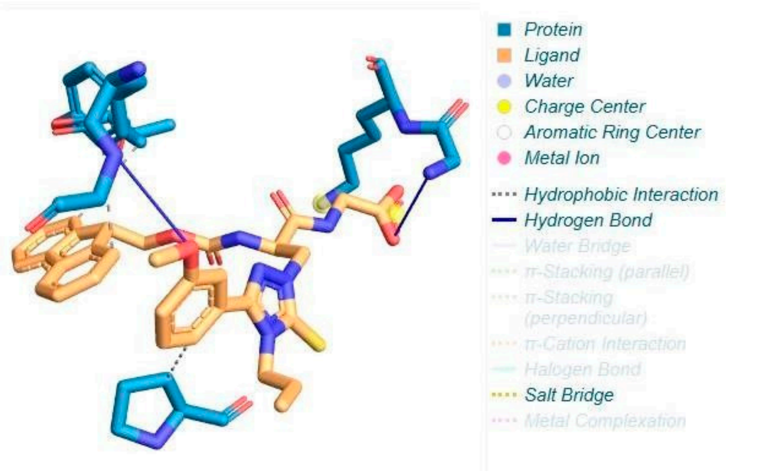

Hydrophobic Interactions

Index	Residue	AA	Distance	Ligand Atom	Protein Atom
1	68A	TYR	3.96	3821	157
2	231A	PRO	3.98	3819	1456
3	377A	ILE	3.91	3820	2630

Hydrogen Bonds

Index	Residue	AA	Distance H-A	Distance D-A	Donor Angle	Protein donor?	Side chain	Donor Atom	Acceptor Atom
1	69A	GLY	3.40	3.77	104.86	✔	✖	160 [Nam]	3810 [O3]
2	93A	GLY	3.07	3.78	130.67	✔	✖	365 [Nam]	3800 [O.co2]

Salt Bridges

Index	Residue	AA	Distance	Protein positive?	Ligand Group	Ligand Atoms
1	94A	LYS	4.81	✔	Carboxylate	3798, 3800

Fluconazole-protein complex

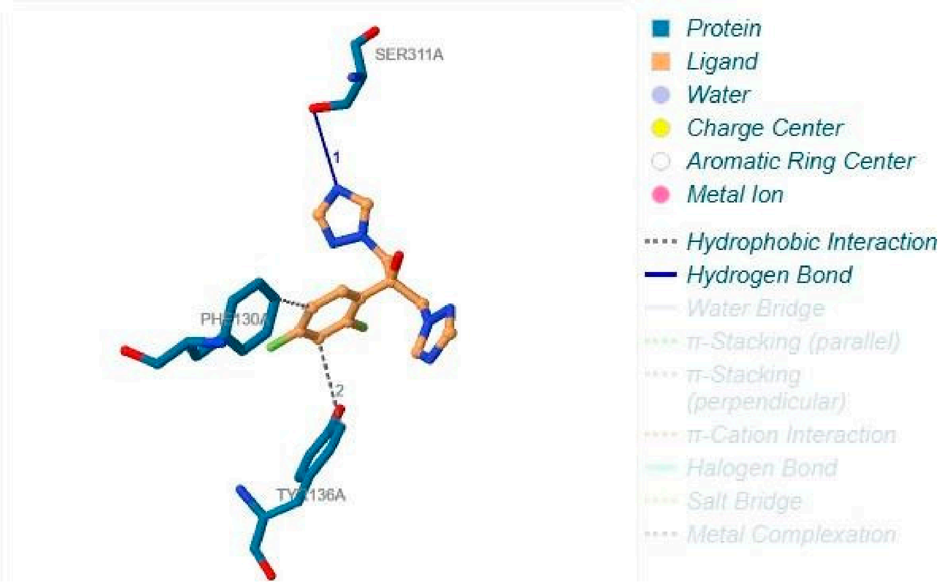

Hydrophobic Interactions ····

Index	Residue	AA	Distance	Ligand Atom	Protein Atom
1	130A	PHE	3.18	3813	657
2	136A	TYR	3.93	3812	706

Hydrogen Bonds —

Index	Residue	AA	Distance H-A	Distance D-A	Donor Angle	Protein donor?	Side chain	Donor Atom	Acceptor Atom
1	311A	SER	3.18	3.54	103.39	✔	✔	2099 [O3]	3804 [Nar]

Figure S3. 3D interaction diagrams for the complexes of Comp 3a, Comp 7a, and fluconazole with the protein as obtained using PLIP (Protein-Ligand Interaction Profiler) (accessed on 3 Dec 2024, Dresden, Germany, Website: https://plip-tool.biotec.tu-dresden.de/).
